# *S-RNase* Alleles Associated With Self-Compatibility in the Tomato Clade: Structure, Origins, and Expression Plasticity

**DOI:** 10.3389/fgene.2021.780793

**Published:** 2021-12-06

**Authors:** Amanda K. Broz, Christopher M. Miller, You Soon Baek, Alejandro Tovar-Méndez, Pablo Geovanny Acosta-Quezada, Tanya Elizabet Riofrío-Cuenca, Douglas B. Rusch, Patricia A. Bedinger

**Affiliations:** ^1^ Department of Biology, Colorado State University, Fort Collins, CO, United States; ^2^ Department of Biochemistry, University of Missouri, Columbia, MO, United States; ^3^ Departamento de Ciencias Biológicas y Agropecuarias, Universidad Técnica Particular de Loja, Loja, Ecuador; ^4^ Center for Genomics and Bioinformatics, Indiana University, Bloomington, IN, United States

**Keywords:** self-incompatibility (incompatible), self-compatibility (compatible), mating system transitions, S-RNase, reproductive barriers

## Abstract

The self-incompatibility (SI) system in the Solanaceae is comprised of cytotoxic pistil S-RNases which are countered by S-locus F-box (SLF) resistance factors found in pollen. Under this barrier-resistance architecture, mating system transitions from SI to self-compatibility (SC) typically result from loss-of-function mutations in genes encoding pistil SI factors such as *S-RNase*. However, the nature of these mutations is often not well characterized. Here we use a combination of *S-RNase* sequence analysis, transcript profiling, protein expression and reproductive phenotyping to better understand different mechanisms that result in loss of S-RNase function. Our analysis focuses on 12 *S-RNase* alleles identified in SC species and populations across the tomato clade. In six cases, the reason for gene dysfunction due to mutations is evident. The six other alleles potentially encode functional S-RNase proteins but are typically transcriptionally silenced. We identified three *S-RNase* alleles which are transcriptionally silenced under some conditions but actively expressed in others. In one case, expression of the *S-RNase* is associated with SI. In another case, *S-RNase* expression does not lead to SI, but instead confers a reproductive barrier against pollen tubes from other tomato species. In the third case, expression of *S-RNase* does not affect self, interspecific or inter-population reproductive barriers. Our results indicate that *S-RNase* expression is more dynamic than previously thought, and that changes in expression can impact different reproductive barriers within or between natural populations.

## Introduction

Self-incompatibility (SI) is a genetic mechanism that prevents self-fertilization in numerous plant species, usually by preventing “self” pollen tube germination on stigmas or self-pollen tube growth in styles (De Nettancourt, 1977; Takayama and Isogai, 2005; Fujii et al., 2016). Although relatively few SI systems have been extensively studied, all examined to date contain a complex *S*-locus which encodes both pistil- and pollen-expressed genes that are polymorphic within populations and act to regulate the specificity of SI (Franklin-Tong, 2008; Fujii et al., 2016; Bedinger et al., 2017; Jany et al., 2019; Nasrallah, 2019). The *S*-locus in the Solanaceae contains a single gene encoding a pistil-expressed *S*-locus RNase (S-RNase) and 15–20 genes encoding pollen-expressed *S*-locus F-box proteins (SLFs) (McClure, 2004; Kubo et al., 2015; Li and Chetelat, 2015; Williams et al., 2015; Wu et al., 2020). Each specific combination of *S-RNase* and *SLF* genes at an *S*-locus constitutes a unique *S*-haplotype, and successful mating only occurs between plants with different *S*-haplotypes.

The SI mechanism operating in the Solanaceae is gametophytic, since it depends on post-meiotic pollen-expressed genes, and can be thought of in terms of a barrier-resistance architecture comprised of pistil-side cytotoxic S-RNases (the barriers) and pollen SLFs that act as resistance factors (Bedinger et al., 2017). In styles, S-RNases are secreted into the transmitting tissue and are taken up by growing pollen tubes. Active S-RNases degrade pollen tube RNA, resulting in pollen tube death, unless they are recognized and detoxified by SLF proteins. Under the non-self-recognition model, the constellation of SLFs produced in pollen tubes of each *S*-haplotype can detoxify all S-RNases except the one encoded by their own haplotype (Kubo et al., 2010). Phylogenetic evidence suggests that SI is the ancestral state in the Solanaceae (Allen and Hiscock, 2008; Igic et al., 2008), and there is frequently high conservation in *S-RNase* allele sequences between species (Ramanauskas and Igić, 2017).

Although SI systems enforce outcrossing and thus maintain genetic diversity within populations, mating system transitions from outcrossing to selfing are common evolutionary events (Darwin, 1876; Stebbins, 1974; Coyne and Orr, 2004; Igić et al., 2008; Wright et al., 2013), especially in circumstances where selfing individuals have a reproductive advantage (Baker, 1955; Baker, 1967; Busch and Schoen, 2008; Pannell et al., 2015). Given the barrier-resistance architecture of SI in the Solanaceae, female-side loss-of-function (pistil first) mutations that lead to self-compatibility (SC) are predicted to be more common than male-side gain-of-function mutations (Bedinger et al., 2017). For example, loss of S-RNase function would eliminate the barrier to self-pollen tube growth. These loss-of-function *S-RNase* mutations are codominant in the sense that plants heterozygous for the mutation will exhibit the SC phenotype and only the haplotype with the non-functional *S-RNase* gene will be transmitted in self-pollinations if the suite of pollen SLFs are intact. In this scenario, self-pollen tubes containing the mutant (SC) *S-RNase* haplotype will be successful in self-pollinations, because all non-self S-RNases will be detoxified, whereas pollen tubes harboring a functional SI haplotype will be destroyed by their self S-RNases. In addition, pollen with the SC haplotype can also be successful in outcross pollinations. These characteristics allow SC to rapidly spread to fixation within a population unless the SC phenotype is countered by other detrimental phenotypes (i.e., pollen/seed discounting; inbreeding depression) (Porcher and Lande, 2005; Busch and Delph, 2012). Alternatively, because of the non-self mode of recognition in S-RNase-based SI, male-side loss-of-function mutations in *SLFs* would not result in SC and could make pollen tubes vulnerable to non-self S-RNases. However, *SLF* gain-of-function mutations in that allow for detoxification of a self S-RNase could result in SC, and there is some evidence these types of mutations can occur at low frequency (Tsukamoto et al., 2003b; Kubo et al., 2015; Markova et al., 2017).

In systems of S-RNase-based SI, there is both functional and mechanistic overlap between SI and pollen-pistil incompatibilities in crosses between species that result in interspecific reproductive barriers (IRBs). Both S-RNase and SLFs have been found to play a role in unilateral IRBs, known as unilateral incompatibility (UI, wherein a cross is incompatible in one direction but the reciprocal cross is compatible) and mutations in the genes encoding these SI factors can alter both interspecific and inter-population reproductive barriers (Tovar-Méndez et al., 2014; Li and Chetelat, 2015; Markova et al., 2016; Broz et al., 2017). SI modifier genes, that are not located at the *S*-locus, have also been implicated in both SI and UI. For example, CUL1 is a pollen-expressed factor that is involved in both SI and UI (Li et al., 2010; Li and Chetelat, 2014). The pistil-expressed modifier HT-proteins are required for SI, and contribute to IRBs (Tovar-Méndez et al., 2014; Tovar-Mendez et al., 2017). However, it is important to note that IRBs can be produced by alternative mechanisms (S-RNase-dependent and S-RNase-independent), and recent studies have identified pistil and pollen factors that contribute to S-RNase-independent IRBs (Qin et al., 2018; Qin and Chetelat, 2021). Because mechanisms of SI and IRBs are only partially redundant, it is not possible to predict how mutation of a particular SI factor will affect interspecific pollen tube growth.

The 13-member tomato clade, *Solanum* section Lycopersicon, is particularly amenable to studying mating system shifts from SI to SC, as multiple independent transitions from SI to SC have occurred both in entire species and within populations of SI species. Six of the 13 tomato species (*S. lycopersicum,* *S. pimpinellifolium, S. galapagense, S. cheesemaniae, S. neorickii* and *S. chmielewskii*) are fully SC, and four predominately SI species (*S. pennellii, S. arcanum*, *S. habrochaites* and *S. peruvianum*) contain one or more SC populations. *S. chilense* has two segregating SI/SC populations (www.tgrc.ucdavis.edu ), and the remaining two species (*S. corneliomulleri* and *S. huaylasense*) are fully SI.

In predominately SI wild tomato species, transitions to SC typically occur at species range margins. For example, the migration of *S. habrochaites* northward through the Amotape-Huancabamba Zone, which consists of microhabitats with widely varying altitudes and temperatures (Weigend, 2002; Weigend, 2004), provides a particularly striking example of multiple independent mating system transitions associated with migration and population differentiation (Landis et al., 2021). This is likely because the ability of a plant to reproduce through self-pollination can provide reproductive assurance to small locally adapted populations colonizing new environments (Baker, 1955, Baker, 1967; Pannell and Barrett, 1998; Pannell et al., 2015).

In many plant species, mating system transitions to SC are associated with changes in floral morphology, often referred to as the “selfing syndrome” (Wright et al., 2013). One prominent phenotype associated with selfing syndrome is reduced flower size, which can evolve when the need for pollinator attraction has been abrogated due to high rates of self-pollination. In the tomato clade, the SC species *S. neorickii* exhibits extremely small flowers and is considered to be autogamous (Rick et al., 1976). Differences in both corolla diameter and stigma exsertion have also been identified between SI and SC populations of *S. habrochaites* (Rick et al., 1979; Broz et al., 2017); although SC populations have not been exhaustively examined.

In the tomato clade, transitions to SC can also be associated with changes in IRBs (Covey et al., 2010; Bedinger et al., 2011; Baek et al., 2015; Broz et al., 2017). In general, UI between tomato clade species follows the SI x SC rule wherein SI species reject pollen tubes of SC species, but the reciprocal cross is compatible, resulting in UI (Baek et al., 2015). However, there are exceptions, particularly in SC populations of typically SI species. For example, an SC population of the typically SI species *S. arcanum* shows decreases in pistil-side IRBs compared to its SI relatives, allowing interspecific pollen tubes to penetrate substantially further into the style (Baek et al., 2015). Self-compatible populations of predominately SI *S. habrochaites* can also show weakened pistil-side IRBs, some of which are associated with the loss of specific pistil-side proteins including S-RNase and HT-protein (Broz et al., 2017).

Here, using a combination of transcriptomics, degenerate PCR amplification, phenotyping and analysis of published sequence data, we characterized *S-RNase* alleles associated with SC across the tomato clade (Table 1). The main objectives of this work were to 1) provide a comprehensive survey of newly discovered and previously identified *S-RNase* alleles that are associated with SC in the tomato clade, 2) evaluate RNA and protein expression of SC-associated *S-RNase* alleles that have no apparent sequence defect, 3) identify putative progenitor (functional) *S-RNase* alleles in SI populations and species and to 4) better understand how SC-associated *S-RNase* alleles affect IRBs. We show that, in most cases, the transition to SC is associated with *S-RNase* mutations that either prevent S-RNase production, reduce S-RNase protein activity, or involve the transcriptional silencing of potentially functional *S-RNase* genes. We find that in some but not all cases, these *S-RNase* mutations affect IRBs in addition to mating system.

**TABLE 1:** Comprehensive list of SC species and populations that have been identified in the tomato clade and their associated *S-RNase* alleles. Representative accessions are listed for SC species and for groups of SC *S. habrochaites* populations.^a^ Li and Chetelat, 2015; ^b^This work; ^c^ Broz et al., 2017; ^d^ Kondo et al., 2002b; ^e^ Kondo et al., 2002a; ^f^ Covey et al., 2010; ^g^ Royo et al., 1994a; ^h^ Kowyama et al., 1994; ^i^ Landis et al., 2021; ^j^ Broz et al., 2021; ^k^ Markova et al., 2017; ^l^HT-protein expression was confirmed with an antibody that binds to both HT-A and HT-B; ^amino acid identity (id) or similarity (sim) of available sequences; *inferred from allele testing in *S. chilense* in Igić et al., 2007 or in *S. peruvianum* in Miller and Kostyun, 2011; ** *S. arcanum LpSC* and *S. chmielewskii LcwSRN-1* are 99.3% identical (Supplementary Figure S2); ***20 amino acids available for alignment prior to frame-shift mutation; ^*#*^ 96 amino acids available for alignment prior to nonsense mutation; NT = not tested, NA = not applicable.

SC species	*S-RNase* allele	GenBank	Represent-ative accession	Mutation or expression defect	RNA Y/N	Protein Y/N	*S*-locus Y/NT	Related *S. chilense* allele	Related functional S-RNase (% aa id/sim^)	HTA/HTB
*S. lycopersicum*	*SRN-red* ^a,b^	AC246123.1, XM004229015	Tomato cultivars	Silenced^b^	N^b^	N^d^	Y^a^	*S20*	*S. chilense* S20 (95.5/97.7)	N^d^/N^d^
*S. pimpinellifolium*	*SRN-red*	KJ814947.1	LA1589	NT	NT	N^b^	NT	*S20*	*S. chilense* S20 (95.5/97.7)	NT/NT
*S. galapagense*	*SRN-orange* ^b^	OK091157	LA0317	NT	NT	N^b^	NT	*S20*	*S. chilense* S20 (96.3/98.5)	NT/NT
*S. cheesmanieae*	*SRN-orange* ^b^	OK091158	LA0522	NT	NT	N^b^	NT	*S20*	*S. chilense* S20 (96.3/98.5)	NT/NT
*S. chmielewskii*	*LcwSRN-1* ^e^	AB072477.1	LA1316	Silenced^e^	N^e^	N^e^	Y*	*S11*	*S. chilense* S11 (100/100)**	Y^e^/N^e^
*S. neorickii*	*LpfSRN-1* ^*e*^	AB072475.1	LA1322	Varies: Silenced or low RNase activity^e,b^	Y^e^/N^b^	Y^e^/N^b^	Y^b^	*S1*	*S. peruvianum* SP2 (96.7/98.3)	Y^be^/N^e^
*S. neorickii*	*LpfSRN-2* ^e^	AB072476.1	LA0247	Frame-shift^e^	N^e^	N^e^	Y^b^	*S7*	*S. arcanum* S6 (95/95***)	Y^bl^
**SC Populations**										
*S. pennellii*	NA	NA	LA0716	Deletion^a^	NA	NA	Y^a^	NA	NA	Y^f^/Y^f^
*S. arcanum*	*LpSc* ^g^	Z26581.1	LA2157	Missense, lacks active site histidine^g^	Y^g,h^	Y^g,h^	Y^h^	*S11*	*S. chilense* S11 (99.3/100)**	NT/NT
*S. habrochaites* SC-1	*hab-7* ^i^	OK091159	LA2119	Silenced in SC-1 group with exceptions^b^	N^i^/Y^b^	N^i^/Y^b^	Y^b^	*S32*	*S. peruvianum* S13 (99.5/100)	Y^b^/N^f^
*S. habrochaites* SC-2	*LhgSRN-1* ^e^	AB072478.1	LA0407	Silenced in SC-2 group^b,c,e,f^	N^e,f^	N^c^	Y^b^	*S6*	*S. habrochaites hab-16* (99.5/100)	Y^f^/N^f^
*S. habrochaites* SC-4	*hab-6* ^f,j^	MW183811.1	LA1927	Missense, low RNase activity^f,j^	Y^f,j^	Y^f,j^	Y^j^	*S2*	*S. peruvianum* SP11 (98.3/99.2)	Y^f^/N^f^
*S. habrochaites* SC-5	*hab-8* ^b^	OK091160	LA2101	Nonsense^b^	NT	N^c^	Y*	*S15*	*S. habrochaites hab-14* ^*#*^ (100/100)	Y^b^/N^f^
*S. habrochaites* SC-6	unknown	NA	LA4654	Unknown	NT	N^i^	NT	NA	NA	Y^i^/N^f^
*S. habrochaites* SC-7	*hab-12* ^b^	OK091161	LA2863	Missense, lacks N-gylcosylation sites^b^	Y^b^	Y^b^	Y*	*S18*	*S. habrochaites hab-13* (99.5/100)	Y^b^/N^f^
*S. peruvianum*	unknown^k^	NA	LA4125	Unknown	NT	NT	NT	NA	NA	NT/NT

## Materials and Methods

### Plant Material and Growth

Seeds were acquired from the C.M. Rick Tomato Genetic Resource Center (TGRC) at University of California, Davis (www.tgrc.ucdavis.edu) or collected in Loja Province in Ecuador (denoted as EC collections) in 2014. Representative accessions for all species and populations are listed in Table 1 and refer to the populations used in our study. Details on additional populations used for study of *S. neorickii* can be found in the Section *S-RNase alleles LpfSRN-1 and LpfSRN-2 in SC S. neorickii*, and those for *S. habrochaites* are provided in the Section *SC accessions in S. habrochaites*. All EC collections, excepting EC40, have representative collections at TGRC (EC6 ~ LA2101, EC7 ~ LA2864 and EC10 ~ 2099) and were verified to exist at the same sites in this study. Seed collections of EC populations are housed at the Departamento de Ciencias Biológicas y Agropecuarias, Universidad Técnica Particular de Loja, Loja, Ecuador. Seeds were sterilized according to recommendations from TGRC. For genotyping, seeds were planted in ProMix-HP and grown on a light shelf for 2 weeks. For experiments to assess pollen tube growth or to produce seeds, plants were grown in 4-inch pots containing ProMix-BX under greenhouse conditions (16 h light at 26°C and 8 h dark at 18°C) until they were 6–12 inches tall, then transplanted to outdoor agricultural fields at Colorado State University or placed in a growth chamber (10 h days) as needed to induce flowering. After performing crosses to obtain specific progeny, fruits were allowed to mature on plants for at least 2 months (or until soft and ripe).

### Pollen Tube Growth Assessment and Reproductive Barrier Phenotyping

In a previously uncharacterized accession of SC *S. habrochaites* (LA2863)*,* and for all *S. neorickii* accessions including F1 and F2 cross types (see *S-RNase Alleles LpfSRN-1 and LpfSRN-2 in SC S. neorickii* Section) we performed reproductive phenotyping. Pollen tube growth in styles was assessed as previously described (Covey et al., 2010). Briefly, emasculated flowers were pollinated, and after 48 h pistils were placed in fixative, softened with NaOH, stained using Aniline Blue Fluorochrome and examined with a fluorescence microscope. In field grown plants, inflorescences were covered with mesh bags to prevent pollinators from interacting with flowers to be used in crosses. Interspecific and inter-population barriers were examined using “tester” lines, as described more thoroughly in Broz et al., 2017. Briefly, to test for IRBs, pistils were pollinated using *S. lycopersicum* cultivars VF36, M82 or LA1221 as males. To test for pistil-side inter-population reproductive barriers in *S. habrochaites*, hand pollinations were performed using *S. habrochaites* SC accession LA0407 as male, and to test for pollen-side inter-population reproductive barriers, hand pollinations were performed using SI accession LA1777 as female.

### Stylar Transcriptome Sequencing and Analysis

Transcriptome sequencing was utilized to identify *S-RNase* alleles in SC (LA2119, LA2863), mixed SI/SC (LA 2099, LA 2098, LA2175) and SI (LA2868, LA2864) accessions of *S. habrochaites*. Unpollinated styles from three individuals of each accession were separately collected into RNAlater solution (Qiagen), and total RNA was extracted using the Qiagen RNeasy Plant Mini Kit. Total RNA was submitted to Indiana University’s Center for Genomics and Bioinformatics for cDNA library construction using a TruSeq Stranded mRNA LT Sample Prep Kit (Illumina) following the standard manufacturing protocol. In some cases, RNA from individuals within an accession were pooled, and sequencing of the unfragmented whole transcriptome libraries was performed on an Illumina MiSeq instrument to generate 250bp paired end reads. In all other cases, sequencing was performed using an Illumina NextSeq500 platform with 150 bp cycle module generating 60 bp paired-end reads. After the sequencing run, demultiplexing was performed with bcl2fastq v2.20.0.422. The raw transcriptome data are available on the NCBI SRA database PRJNA310635. Details on data processing, analysis and identification of *S-RNase* sequences are described in Broz et al., 2021. Transcriptome analysis led to the identification of new *S-RNase* alleles *hab-7*, *hab-12*, *hab-13*, *hab-14*, *hab-15*, *hab-16* and *hab-17* (GenBank numbers OK091159, OK091161- OK091166).

### Degenerate PCR to Isolate *hab-8 S-RNase* Allele

A PCR-based strategy devised by Kondo *et al.* (2002a) was used for the isolation of the *S-RNase* allele from *S. habrochaites* accession LA2101. Briefly, we amplified unknown *S-RNase* sequences from the genomic DNA using degenerate primers based on conserved *S-RNase* sequences (Covey et al., 2010) and appropriately sized products were gel purified (Qiagen) and ligated to pJET1.2 (ThermoFisher). Colony PCR was performed, and the resulting PCR products were purified (Zymo) and sequenced (Genewiz). The sequence identified in LA2101 is *hab-8*, GenBank OK091160.

### PCR Amplification and Sequence Analysis

We used PCR amplification and sequencing to obtain the *S-RNase* alleles for *S. galapagense* LA0317 and *S. cheesmaniae* LA0522, to verify alleles from *S. habrochaites* that were identified by transcriptome analysis (see *Stylar Transcriptome Sequencing and Analysis* Section), and to verify previously identified alleles from *S. neorickii.* Genomic DNA was extracted from leaf tissue of seedlings in 200 mM Tris-HCl pH 9.0, 250 mM NaCl, 25 mM EDTA, and 1% SDS, followed by precipitation in isopropanol. All primers are listed in Supplementary Table S1, including primers designed to amplify specific *S-RNase* alleles. PCR was performed using EconoTaq Plus Green Mastermix (Lucigen). Genomic DNA quality was assessed by amplifying single copy control genes (Supplementary Table S1). For genotyping, PCR products were analyzed on 1.2% agarose gels. For sequencing, PCR products were purified (Zymo) and both strands of amplicons were sequenced (GeneWiz). Genomic DNA and deduced amino acid sequences were aligned using MUSCLE (http://www.ebi.ac.uk/Tools/msa/muscle/ (Madeira et al., 2019). Signal peptide predictions were made using TargetP http://www.cbs.dtu.dk/services/TargetP/ and N-glycosylation site predictions were made using NetNGlyc 1.0 http://www.cbs.dtu.dk/services/NetNGlyc/.

### Reverse Transcriptase-PCR

*Solanum neorickii LpfSRN-1* expression was tested using RT-PCR. Total RNA was purified from both mature pistils and leaves using a Qiagen RNeasy Plant Mini Kit and treated with a Qiagen RNase-Free DNase Kit. First strand cDNA templates were synthesized using a Bio-Rad iScript cDNA Synthesis Kit (http://www.bio-rad.com) using cycling conditions of 25°C for 5 min, 40°C for 30 min, and 85°C for 5 min. EconoTaq plus Green Mastermix (Lucigen) was used to amplify cDNA with the *LpfSRN-1* (test) and *CAC* (positive control) primer sets (Supplementary Table S1). RT-PCR products were run on a 1.2% agarose gel to examine expression levels.

### Immunostaining of Stylar S-RNase Proteins

Immunostaining was performed for all red and orange fruited species (see *S-RNase Alleles in Four SC Red/Orange-Fruited Tomato Species* Section), all *S. neorickii* accessions and cross types (see *S-RNase Alleles LpfSRN-1 and LpfSRN-2 in SC S. neorickii* Section), and for selected *S. habrochaites* accessions that had not been previously analyzed for S-RNase protein expression (see *SC Accessions in S. habrochaites* Section). Stylar proteins were extracted from at least 10 mature, post-anthesis unpollinated styles to test for S-RNase expression. Weighed styles were homogenized in 2x SDS buffer (0.125 M Tris-HCl pH 6.8, 4% SDS, 20% glycerol, 50 mM dithiothreitol, and 0.01% Bromophenol blue) at 10 µL per mg fresh weight. After grinding styles in the buffer, samples were heated for 5 min at 90°C and centrifuged at 14,000 g for 10 min. The supernatant was collected and frozen until use.

For each individual tested, protein extract equivalent to 0.2 mg fresh weight (unless otherwise noted) was separated by electrophoresis, blotted, and immunostained as previously described (Covey et al., 2010). tSRNC2 antibodies raised against the S-RNase conserved C-2 domain, were used as probes for S-RNase (Chalivendra et al., 2013), and those raised against a conserved peptide in HT-A and HT-B were used as probes for HT-protein (Broz et al., 2017).

### Segregation Analysis for *S*-Locus Localization

Since there are numerous *RNase* genes in plant genomes that resemble *S-RNase* genes, we assessed whether alleles from *S. neorickii* (*LpfSRN-1*, *LpfSRN-2*) and *S. habrochaites* (*LhgSRN-1* and *hab-7*) segregated as would be predicted for a gene at the *S*-locus. We crossed females that were homozygous for well-characterized loss-of-function *S-RNase* alleles with males that were heterozygous for the *S-RNase* allele being tested and an *S-RNase* allele known to be at the S-locus. Allele-specific PCR was used to identify *S-RNase* sequences in progeny, including the expected female allele as a DNA quality control. If the male allele being tested is at the *S*-locus, we expect that it would never be inherited with the male allele known to be at the *S*-locus. The Freeman Halton extension of Fishers exact test was used to determine whether observed (progeny genotype) values differed from expected values if the tested allele was at the *S*-locus (0AB:1A:1B) or was not linked to the *S*-locus (2AB:3A:3B).

### Floral Characters in SI and SC *S. habrochaites* Populations

The transition to SC is often correlated with reductions in flower size, and we wanted to assess this trait in populations of *S. habrochaites*. Flower size was measured with digital calipers *in situ* in Ecuador, but to increase the accuracy of measurements, flowers from plants grown in a common garden at Colorado State University in the summer of 2016 were first preserved using clear packing tape as previously described (Spooner and Van Den Berg, 2001). The reproductive whorls were removed by snipping them at their base using forceps, and the corolla lobes were rolled out to stick to the tape, with the calyx removed. All open flowers of three separate inflorescences were scanned at high resolution (1200dpi) and measured digitally using ImageJ (Schneider et al., 2012). Measurements included petal length (A), inter-petal distance (B), width (C), sepal length (E), anther length (F), and stigma exsertion (G). Corolla area was approximated by calculating the area of a 5-pointed star [5AB * sin (36°)], where A = petal length and B = inter-petal distance.

A mixed model was used to detect significant differences between collection regions while accounting for sources of environmental variation and experimental blocks. Field designation (north or south plot), field position (row and column), flower collection date, and days post anthesis (day 0, 1, etc.) were used as random effects to detect significant (*p* < 0.05) differences between geographical regions (modeled as a fixed effect) for each variable. Generalized linear models were similarly used to detect significant differences between regions of collection sites for the other morphological observations (both *in situ* and common garden).

## Results

### *S-RNase* Alleles in SC Species

#### *S-RNase* Alleles in Four SC Red/Orange-Fruited Tomato Species

**FIGURE 1:**
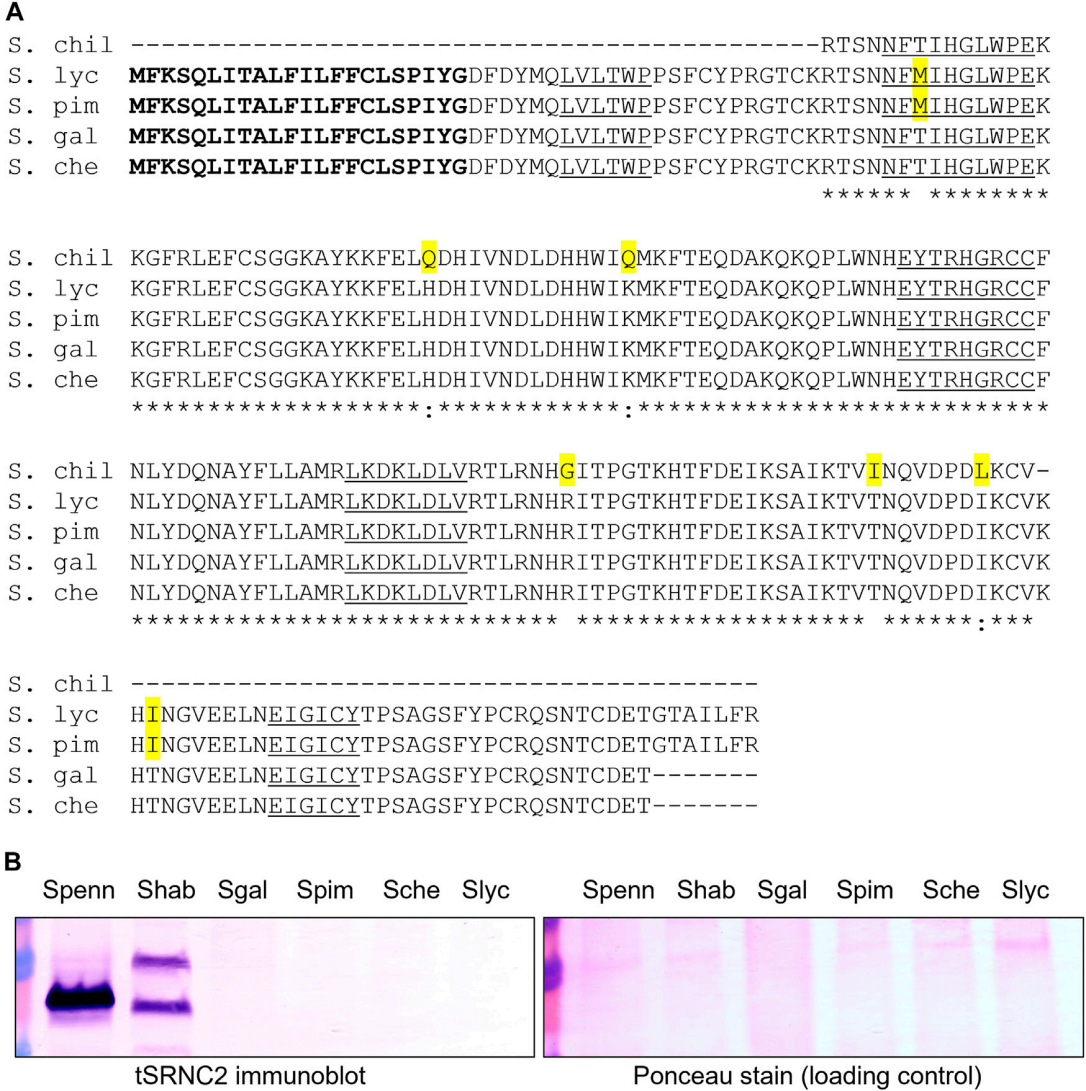
S-RNase alignment and immunoblot from four red/orange-fruited tomato species. **(A)** alignment of deduced amino acid sequences S. chil = *S. chilense* S20 S-RNase, partial sequence (Igić et al., 2007) GenBank EF680098, S. lyc = *S. lycopersicum* cultivar Heinz 1706 (Li and Chetelat, 2015) Sol Genomics Network (https://solgenomics.net) Solyc01g055200.1, S. pim = *S. pimpinellifolium* accession LA1589 coding sequences Sol Genomics Network Sopim01g055200.0.1, S. gal = *S. galapagense* accession LA0317 PCR product, this study, GenBank OK091157, S. che = *S. cheesmaniae* accession LA0522 PCR product, this study, GenBank OK091158. The predicted signal peptide is bolded, amino acid substitutions are highlighted in yellow and conserved S-RNase sequences C1-C5 are underlined. Asterisks indicate conservation between all sequences. **(B)** Immunoblot of stylar extracts using the tSRNC2 antibody raised to the conserved C2 region of S-RNases. Ponceau Stain of the membrane is shown as a loading control. Extracts from two SI plants were run as positive controls. Spenn = SI *S. pennellii* LA1340, Shab = SI *S. habrochaites* LA1777, Sgal = SC *S. galapagense* LA0438, Spim = SC *S. pimpinellifolium* LA1245, Sche = SC *S. cheesmaniae* LA0522, Slyc = SC *S. lycopersicum* LA4444.

Four of the six SC tomato clade species group in a subclade of closely related species that produce red or orange fruits: *S. lycopersicum*, *S. pimpinellifolium*, *S. galapagense*, and *S. cheesmaniae.* The *S*-locus of *S. lycopersicum* is one of the few *S*-loci in the Solanaceae to be completely sequenced (Sato et al., 2012, https://solgenomics.net). Li and Chetelat (2015) analyzed the *S*-locus of cultivated tomato and reported the presence of a single *S-RNase*-related sequence associated with a cluster of *SLF* genes in the pericentric region of Chromosome 1, as predicted for the *S*-locus in *Solanum*. Originally, the *S-RNase*-like sequence was referred to as a pseudogene with a 93-bp insertion, and it was proposed that this insertion could explain the lack of RNase activity in *S. lycopersicum* styles (Kondo et al., 2002b). However, a closer examination of the sequence reveals that the putative insertion is actually the characteristic single intron found in all Solanaceous *S-RNase* genes (Supplementary Figure S1). Similar sequences are found in all four members of the SC red/orange-fruited subclade (Figure 1A; Supplementary Figure S1), suggesting that this allele became fixed in a common ancestor to the group.

The predicted amino acid sequences of the encoded S-RNases in the red/orange-fruited species (Figure 1A) contain the five known conserved sequences C1-C5 in known S-RNases and are closely related to the known functional S20 S-RNase in *S. chilense* (Igić et al., 2007). There are two non-conservative differences between the pair of red-fruited species (*S. lycopersicum* and *S. pimpinellifolium*) compared to the pair of orange-fruited species endemic to the Galapagos Islands (*S. galapagense* and *S. cheesmaniae*). Given these differences, we refer to the *S-RNase* alleles as *SRN-red* for the allele in red-fruited species and as *SRN-orange* in the orange-fruited species (Table 1). One of the non-conservative differences between the encoded S20 S-RNase in *S. chilense* and the S-RNase encoded in red-fruited species is a Thr→ Met substitution in the C2 conserved domain that would eliminate an N-glycosylation site that is highly conserved in Solanaceae S-RNases, and which may reduce RNase function but should not prevent expression (Williams et al., 2015). These results suggest that the *S-RNase*-like sequences at the *S*-locus in these species could be expressed and, at least in the orange-fruited species, encode a potentially functional S-RNase. Previous work has indicated that styles of *S. lycopersicum* do not express S-RNase protein (Tovar-Méndez et al., 2014). We confirmed this and found that styles of the other red and orange fruited species also do not express S-RNase protein (Figure 1B). Further, transcriptome data collected on Sol Genomics (https://solgenomics.net/) and stylar RNAseq studies of *S. lycopersicum* (Pease et al., 2016) show no expression of the *S-RNase* gene. At this time, there is no clear explanation for the lack of *SRN-red* or *SRN-orange* expression, and therefore we classify these alleles as silenced (Table 1). The red- and orange-fruited species lack HT expression in addition to S-RNase expression (Kondo et al., 2002b), and lack IRBs, which can be partially restored by the transgenic introduction of functional *S-RNase* and *HT* genes (Tovar-Méndez et al., 2014).

#### *S-RNase* Allele *LcwSRN-1* in SC *S. chmielewskii*

In addition to the four red/orange fruited tomato species, there are two additional SC species that group within a subclade known as the Arcanum group, which contains three species: SI *S. arcanum*, SC *S. chmielewskii* and SC *S. neorickii*. Recent data have shown that while both SC species are derived from SI *S. arcanum*, they are independently derived from distinct geographical subsets of *S. arcanum* (Florez-Rueda et al., 2021). The single known *S-RNase* allele in *S. chmielewskii*, *LcwSRN-1* (after the previous species name, *Lycopersicum chmielewskii*), is not expressed at the RNA level (Kondo et al., 2002a), and the transition from SI to SC in *S. chmielewskii* likely followed the typical pistil first pattern with a loss of S-RNase function (Markova et al., 2017). The predicted amino acid sequence of the encoded LcwSRN-1 protein is 100% identical to that of the known functional S11 S-RNase of *S. chilense* in the aligned region (Igić et al., 2007); Supplementary Figure S2). Thus, *LcwSRN-1* likely represents another example of a potentially functional *S-RNase* allele that is transcriptionally silenced by an as yet unknown mechanism (Table 1).

#### *S-RNase* Alleles *LpfSRN-1* and *LpfSRN-2* in SC *S. neorickii*

*S. neorickii* is the other SC species in the Arcanum group. Kondo *et al.*, 2002a isolated two *S-RNase* alleles from *S. neorickii*, *LpfSRN-1* and *LpfSRN-2* (after the previous species name, *Lycopersicum parviflorum*). *LpfSRN-2* is closely related to functional *S. arcanum* allele S6 (Royo et al., 1994a) but is non-functional due to a 1-bp insertion causing a frame shift that results in a premature stop codon (Supplementary Figure S3).

In contrast, *LpfSRN-1* has no obvious defect in its coding region (Kondo et al., 2002a) and the predicted protein is closely related to that of a known functional *S. peruvianum* S-RNase *SP2* [(Miller and Kostyun, 2011); Supplementary Figure S4]. *LpfSRN-1* is transcribed and translated in *S. neorickii* accession LA1322, although no RNase activity above background is detected in styles (Kondo et al., 2002a). Although it was initially assumed that SC in this species is due to the loss of S-RNase expression or function (Kondo et al., 2002a), recent evidence suggests SC may have resulted from the acquisition of a pollen-expressed *SLF23* gene whose encoded protein can detoxify the self LpfSRN-1 RNase (Markova et al., 2017). However, because both pollen and pistil SI specificity genes have undergone mutation and are fixed in this species, it is difficult to know which mutation came first.

**FIGURE 2:**
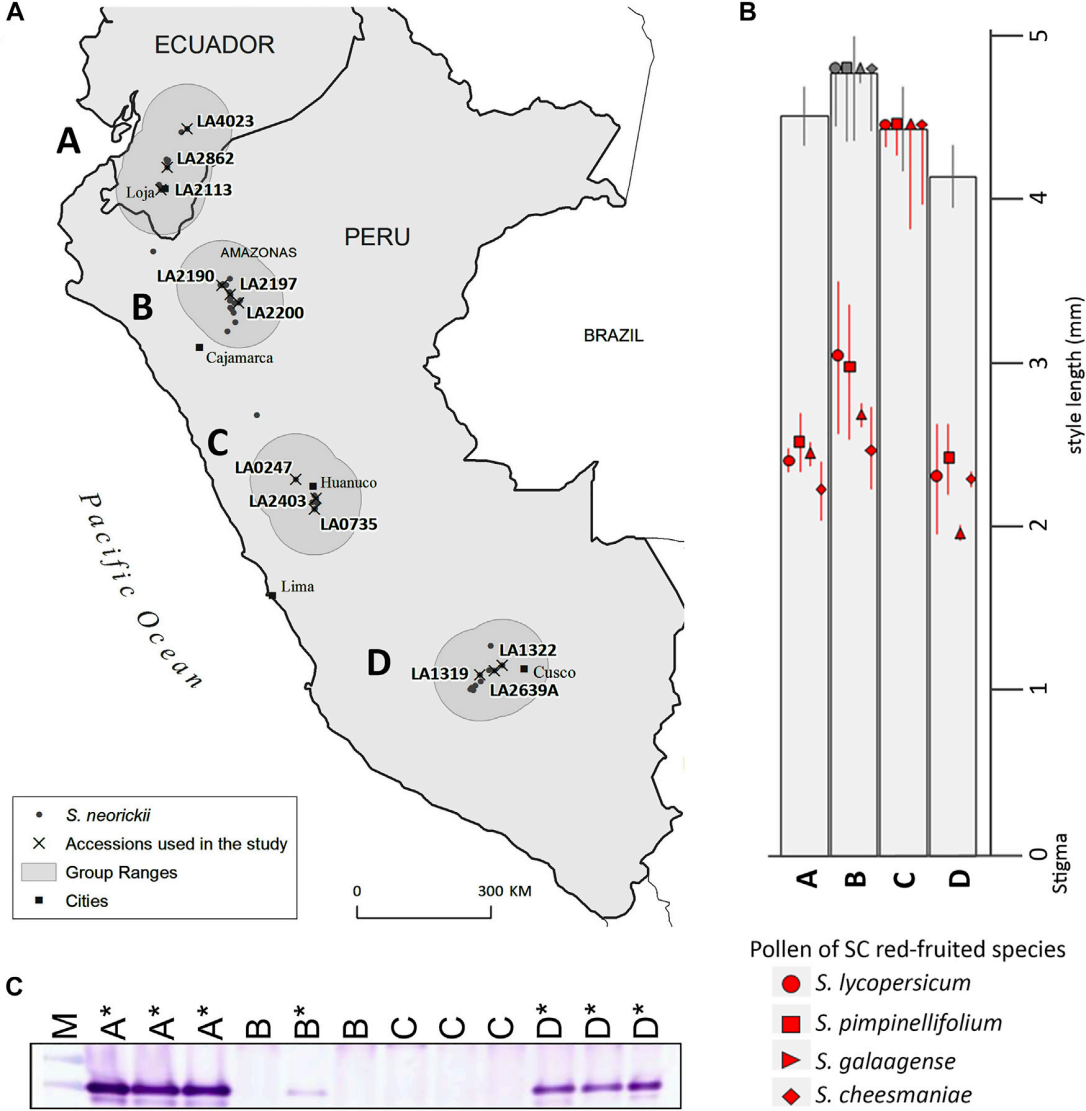
Distribution, IRBs and S-RNase expression in *S. neorickii* geographic groups A-D**. (A)** Accessions used in this study are grouped according to geographical location (north to south) and range (shaded regions) within the species distribution. **(B)** Red/orange species pollen tube lengths 48 h post-pollination in *S. neorickii* styles are shown in millimeters and include the average length of the majority of pollen tubes (species symbols) and standard deviation (bars). Pollen tubes grew to ovaries in some individuals of geographic group B (gray symbols), but not in others (red symbols). **(C)** Immunoblot of stylar extracts using the tSRNC2 antibody raised to the conserved C2 region of S-RNases. Sample order is M, molecular weight marker; LA4023 (group A); LA2862 (A); LA2113 (A); LA2190 (B); LA2197 (B); LA2200 (B); LA0247 (C); LA0247 (C); LA0735 (C); LA1322 (D); LA2639A (D); LA1319 (D). * = accessions/individuals in which pollen tubes from red-fruited species are rejected in styles (presence of IRBs). The expression of S-RNase in Group B LA2197 was confirmed in a separate blot using a greater volume of extract (not shown).

Previous work demonstrated that, although all *S. neorickii* accessions are SC, some accessions had functional IRBs (Baek et al., 2015). We therefore hypothesized that although LpfSRN-1 S-RNase cannot function in SI, it may still be able to function in interspecific pollen tube rejection. Because *S. neorickii* is distributed into four distinct geographic groups within its range [(Figure 2A) (groups A (Ecuador), B (Amazonas, Peru), C (Huánco, Peru) and D (Cusco and Apurimac, Peru)], we selected three accessions from each geographic group for further studies of IRBs.

We assessed whether the two different alleles previously identified in *S. neorickii* (*LpfSRN-1* and *LpfSRN-2*) are associated with different populations in the geographic distribution of the species using allele-specific PCR (Supplementary Figure S5). We found the *LpfSRN-1* allele in all accessions tested, consistent with the findings of Markova *et al.* (2017). However, we found the *LpfSRN-2* allele in group A and C accessions, but not in all B and D group accessions. Upon sequencing, all *LpfSRN-2* alleles in positive accessions that were tested (data not shown) contained the same loss-of-function insertional mutation that was previously reported (Kondo et al., 2002a).

Because *S. neorickii* is highly autogamous, finding two *S-RNase* alleles in a presumably “heterozygous” state in multiple individuals from each of the A and C accessions was surprising. We hypothesized the two *S-RNase* alleles are linked to each other, putatively the result of transposition and/or genetic exchange near the *S*-locus as has been documented in Petunia (Wu et al., 2020). We tested for linkage of the two *S. neorickii* S-RNase alleles to each other and for *S*-locus localization using segregation analysis. Plants that contained the two *S. neorickii S-RNase* alleles (*LpfSRN-1* and *LpfSRN-2*) and *hab-7*, an *S-RNase* allele known to be at the *S*-locus (shown below), were used as males in crosses with female plants that were homozygous for a known *S-RNase* allele (*SRN-red* or *LhgSRN-1*, Supplementary Table S2). By analyzing *S-RNase* alleles in the progeny of this cross, we found that the two *S. neorickii* alleles were always inherited together, and never separately. Further, the two *S. neorickii* alleles were never inherited with the *hab-7* allele in progeny plants. These results are consistent with the two *S. neorickii* alleles being linked to each other and with these alleles being located at, or near, the *S*-locus (Supplementary Table S2).

We examined variation of IRBs in *S. neorickii* by pollinating pistils of accessions from each geographic group with pollen from red -fruited species and evaluating pollen tube growth in styles (Figure 2B; Supplementary Figure S6). We found that styles of geographic groups A and D accessions reject interspecific pollen tubes (IRBs present), whereas styles of group C accessions do not (IRBs absent), and styles of group B accessions varied depending on the individual being tested (IRBs segregating). Since previous work had demonstrated that S-RNase expression (with HT-protein) could constitute an IRB acting on pollen of red-fruited species (Tovar-Méndez et al., 2014) we next tested the same accessions for expression of S-RNase and HT by immunoblotting stylar extracts. Previously HT-A (but not HT-B) was identified in *S. neorickii* LA1322 (Group D) (Kondo et al., 2002a). We probed stylar extracts of *S. neorickii* accessions with an antibody designed to a peptide present in both HT-proteins and show that all accessions tested expressed HT-protein (Supplementary Figure S7). We found that accessions that were able to reject interspecific pollen also expressed S-RNase, whereas those that lacked IRBs did not express S-RNase at the mRNA or protein levels (Figure 2C, Supplementary Figure S8). Since the *LpfSRN-1* allele is silenced in some accessions and expressed in others, although no RNase activity is detected in styles (Kondo et al., 2002a), we classified this allele as both transcriptionally silenced and low S-RNase activity when expressed (Table 1).

**FIGURE 3:**
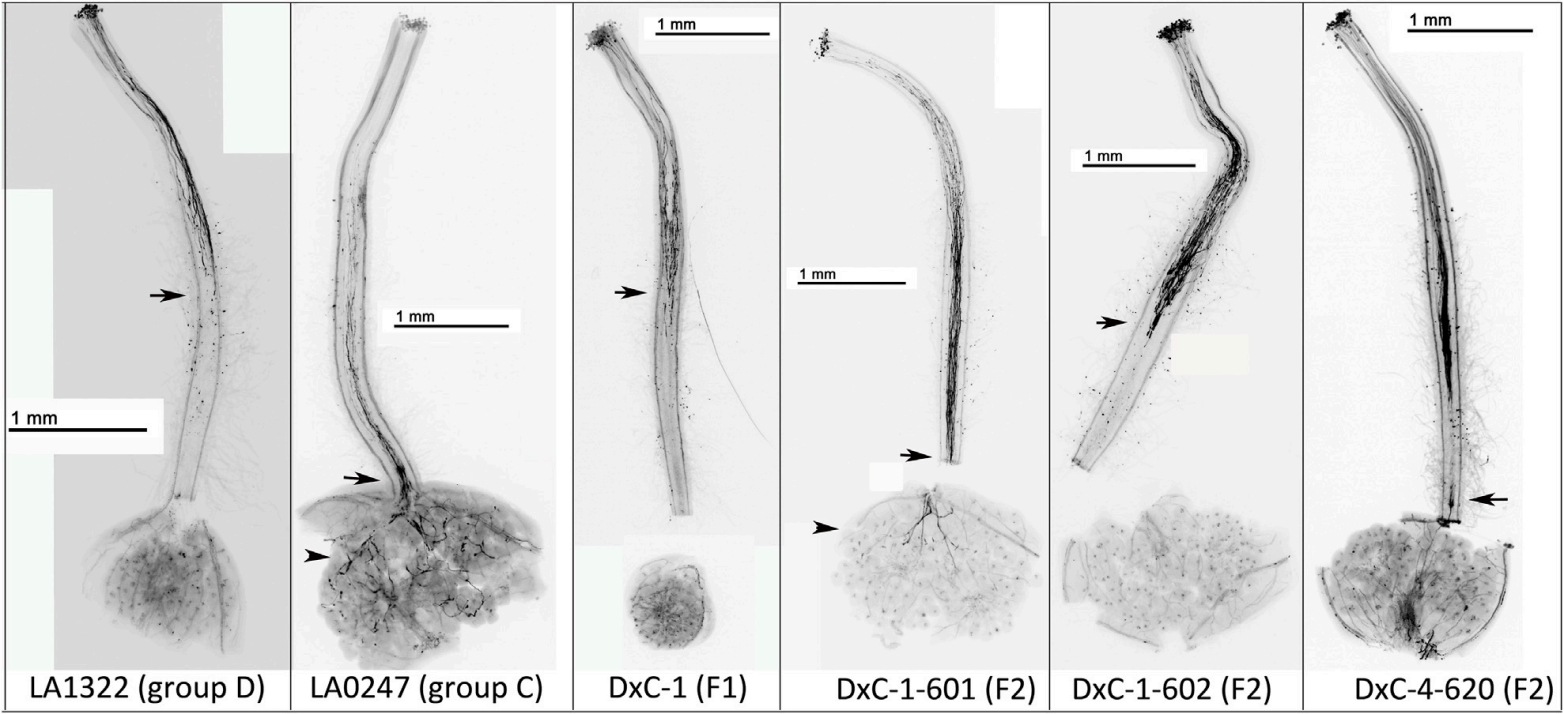
IRBs in *S. neorickii* geographic groups D and C, F_1_ and F_2_ plants. Representative images of crosses in the F2 plants with pollen from red-fruited species (*S. lycopersicum* or *S. pimpinellifolium*). Plants used as the female in crosses are listed in the figures. Arrow indicates the point at which the majority of rejected pollen tubes stop growing and arrowhead indicates pollen tubes in the ovaries.

To determine whether IRBs were dominant, we crossed a group C accession lacking IRBs (LA0247) and a group D accession possessing IRBs (LA1322). In all F_1_ hybrid plants tested, all progeny expressed S-RNase protein and rejected interspecific pollen (Figure 3; Supplementary Table S3, Supplementary Figure S9). Four different F_1_ plants were self-pollinated and F_2_ progeny were phenotyped for both IRBs and S-RNase expression. We found that all F_2_ plants that reject interspecific pollen tubes (possess IRBs) also express S-RNase protein (17/49, Supplementary Table S3; Supplementary Figure S9). However, a significant number of F_2_ plants that accept interspecific pollen tubes (lack IRBs) also express S-RNase protein (12/49) (Supplementary Table S3; Supplementary Figure S9). These results suggest that expression of LpfSRN-1 S-RNase protein is necessary, but is not sufficient, for interspecific pollen tube rejection and therefore that another pistil factor is likely required for the observed IRBs.

### *S-RNase* Alleles in SC Populations of SI Species

#### *S. pennellii* SC Accession LA0716 *S-RNase* Deletion

*S. pennellii* is a generally SI species, but a small number of accessions identified at the southern range margin exhibit SC. The best characterized of these is SC accession LA0716, which has been extremely useful for both basic research and applications to agriculture. For example, the ease of producing fertile F1 hybrids (Rick, 1960) allowed the construction of introgression lines (Eshed and Zamir, 1995) that have been used to generate a detailed physical map of the tomato genome and to isolate important agronomic factors (Lippman et al., 2007). The SC trait of this accession was also essential for the generation of the first complete genome sequence of a wild tomato species not closely related to the cultivated species (Bolger et al., 2014). S-RNase is undetectable in this accession either by activity (Covey et al., 2010) or by immunostaining (Chalivendra et al., 2013). Analysis of the *S*-locus in LA0716 failed to identify even a remnant *S-RNase* gene, suggesting that the gene has been deleted (Li and Chetelat, 2015; Table 1). It is thought that the complete lack of S-RNase caused a pistil first mating system transition in this accession, since male components that contribute to both SI and IRBs are active (Li and Chetelat, 2010; Li and Chetelat, 2015). Because LA0716 lacks S-RNase but exhibits robust IRBs (Baek et al., 2015), this accession has also been extremely useful for identifying genes involved in S-RNase-independent IRBs (Tovar-Méndez et al., 2017; Qin et al., 2018; Qin and Chetelat, 2021).

#### *S. arcanum* SC Accession LA2157 *LpSc* Allele

In *S. arcanum*, a single SC accession (LA2157) has been identified in this otherwise SI species. The *S-RNase* allele in LA2157 *LpSc* (after the previous species name, *Lycopersicum peruvianum*) is expressed at the protein level but a missense mutation eliminates a histidine residue essential for RNase activity (Royo et al., 1994b; Table 1). The protein encoded by the *LpSc* allele segregates with the SC phenotype, indicating that the allele resides at the *S*-locus and is responsible for the SC phenotype (Royo et al., 1994a). Except for the single amino acid substitution in the active site, the amino acid sequence of LpSc S-RNase is identical to both LcwSRN-1 (Markova et al., 2017) and functional *S. chilense* S11 S-RNase (Supplementary Figure S2). The transition from SI to SC in this *S. arcanum* accession likely followed the typical pistil first pattern of mutations with a loss of pistil S-RNase expression/activity (Markova et al., 2017). Pistil-side IRBs are greatly weakened in LA2157 compared to SI *S. arcanum* accessions (Baek et al., 2015; Tovar-Méndez et al., 2017) suggesting that loss of S-RNase activity causing a mating system transition to SC also affects IRBs.

#### SC Accessions in *S. habrochaites*

**FIGURE 4:**
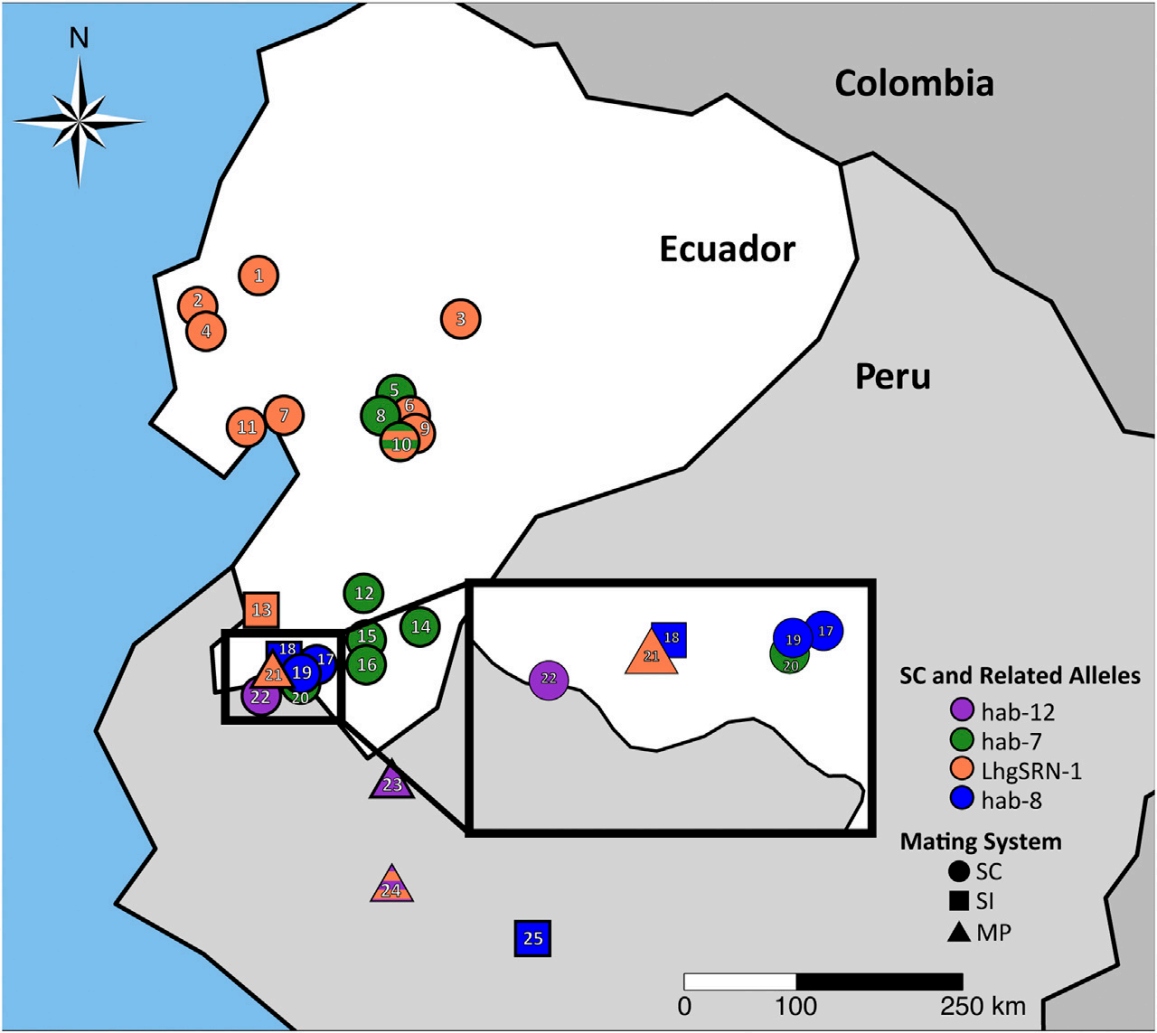
SC-associated *S-RNase* alleles and ancestral SI-associated *S-RNase* alleles in *S. habrochaites* at the northern species margin. Alleles associated with self-compatibility (SC) are found in SC populations (circles), and their related putatively ancestral alleles are found in segregating SI (square) or SI/SC mixed populations (MP, triangles). Colors represent differentiated populations as described in Landis et al., 2021. For a key to accessions displayed see Supplementary Table S4. Orange = *LhgSRN-1* or *LhgSRN-1-like* alleles (SC-2), green = *hab-7* allele (SC-1), striped orange/green = segregating *LhgSRN-1* and *hab-7* alleles in a region of SC-1/SC-2 hybridization, blue = *hab-8* or *hab-8-like* alleles (SC-5), purple = *hab-12* or *hab-12-like* alleles (SC-7). *LhgSRN-1-like* alleles are also found in SI or MP populations in central Peru (Supplementary Table S4).

Remarkably, SC has arisen at least six times in the generally SI species *S. habrochaites* (Table 1). Five of the six known SI →SC transitions occurred in Ecuador at the northern species margin, and the SC-associated *S-RNase* alleles found in these SC accessions were likely derived from those present in ancestral SI populations in the region near the Ecuador-Peru border (Figure 4). The SC accessions of *S. habrochaites* have been categorized into groups (SC-1 to SC-6) based on distinct reproductive phenotypes (Broz et al., 2017; Broz et al., 2021; Landis et al., 2021) and specific *S-RNase* alleles (Table 1). Recent studies have demonstrated that these SC groups also display population differentiation (Landis et al., 2021).

Since reduced flower size is a character often associated with the selfing syndrome that can result from mating systems transitions to SC (Sicard and Lenhard, 2011; Wright et al., 2013), we measured corolla area across *S. habrochaites* SC and SI accessions in Ecuador (Supplementary Figure S10). Overall, we found that SC *S. habrochaites* accessions have not undergone floral size reduction, with the exception of SC-2 accessions at the far northern species margin, consistent with previous reports (Rick et al., 1979; Broz et al., 2017). However, we found a significant increase in the number of floral buds per inflorescence in SI versus SC populations (Supplementary Figure S10), which could potentially increase pollinator attraction in obligate outcrossers. We next examined the structure, origin and expression of *S-RNase* alleles involved in mating system transitions to SC in *S. habrochaites*.

##### The *hab-8 S-RNase* Allele

**FIGURE 5:**
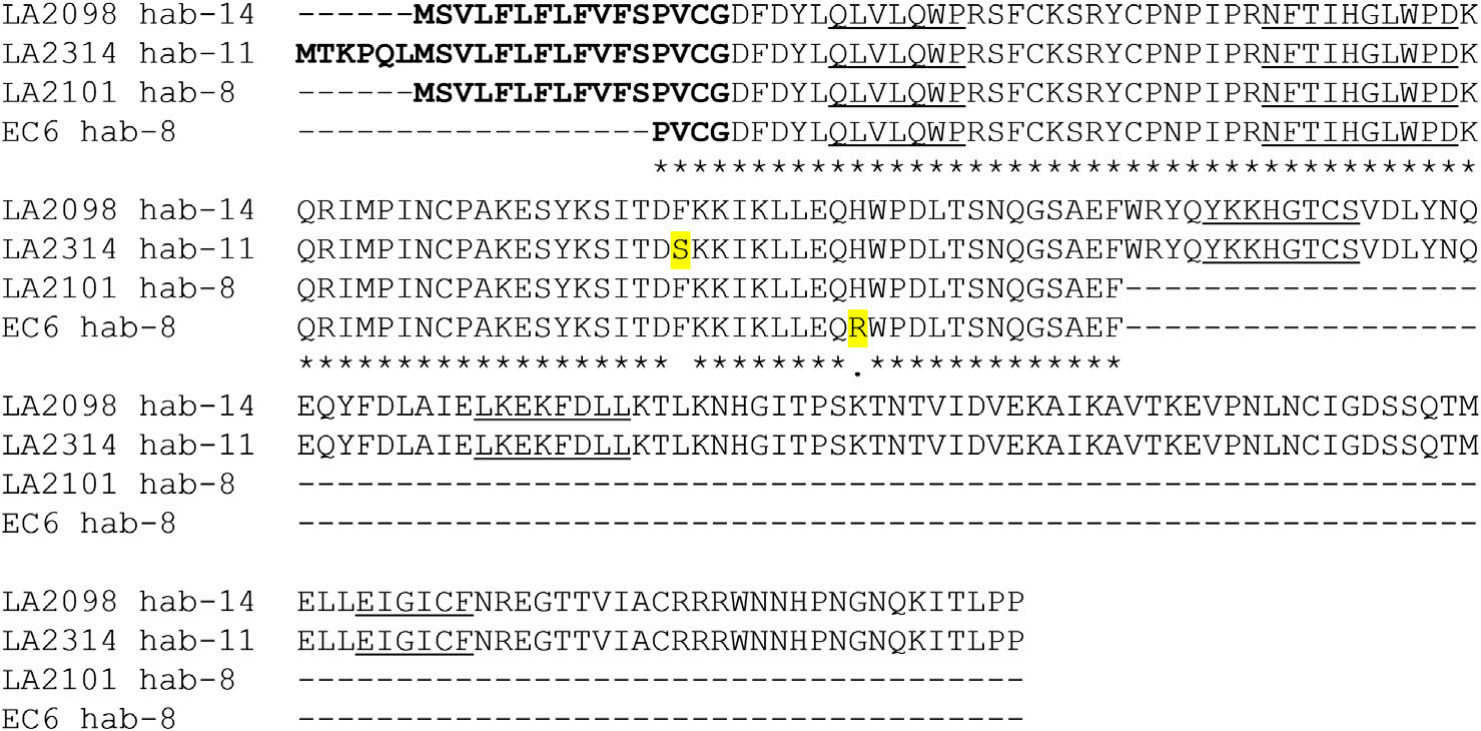
Amino acid sequence of *S. habrochaites* SC-associated hab-8 S-RNase aligned with amino acid sequence of SI-associated hab-14 and hab-11 S-RNases. Predicted amino acids of hab-8 S-RNase (GenBank OK091160) from SC accessions LA2101 and EC6 are aligned with predicted hab-14 sequences (GenBank OK091163) from SI individuals from mixed SI/SC accession LA 2098 (an identical sequence was recovered from SI accession LA2864, not shown) and hab-11 in LA2314 (identical to partial codon sequence of hab-11 in accession LA0094, GenBank MW183817, not shown). The predicted signal peptide is bolded, amino acid substitutions are highlighted in yellow and conserved sequences C1-C5 are underlined. Asterisks indicate conservation between all sequences.

The newly discovered *hab-8 S-RNase* allele was identified in accession LA2101, collected in San Pedro de Cariamanga, Ecuador, in 1980 and was also found in wild populations at the same site in 2014 (site EC6, Figure 4). Transcripts of *hab-8* were not detected using RNA-seq, and S-RNase protein is not detected by immunoblotting (Broz et al., 2017). The predicted protein encoded by the *hab-8* allele is truncated due to a G→ A transition that creates a premature stop codon, i.e., a nonsense mutation (Figure 5; Supplementary Figure S11). The encoded hab-8 S-RNase is identical prior to the premature stop codon to that encoded by the *hab-14 S-RNase* allele segregating in SI accession LA2864 and in the mixed SI/SC accession LA 2098, both only ∼40 km from the SC accessions containing *hab-8* (Figure 4; Supplementary Table S4). In turn, the *hab-14* allele encodes an S-RNase that is highly similar (a single non-conservative amino acid substitution) to that encoded by the previously reported *hab-11* allele found in SI plants in accessions LA0094 and LA2314 from Peru (Broz et al., 2021).

SC accessions near San Pedro de Cariamanga were designated as being group SC-5, and population structure analysis indicates a close relationship of group SC-5 with SI accessions in southern Ecuador (Landis et al., 2021; Figure 4). Previous studies have shown that SC-5 plants do not express S-RNase but do retain S-RNase-independent IRBs (Broz et al., 2017; Landis et al., 2021). We found that pistil-expressed HT-A, a protein involved in both SI and IRBs (Tovar-Méndez et al., 2014; Tovar-Méndez et al., 2017; Table 1) appears to be functional based on sequence analysis of RNA-seq data (Supplementary Figure S12) and is expressed at the protein level (Broz et al., 2017) in SC-5 accessions.

##### The *hab-12 S-RNase* Allele

**FIGURE 6:**
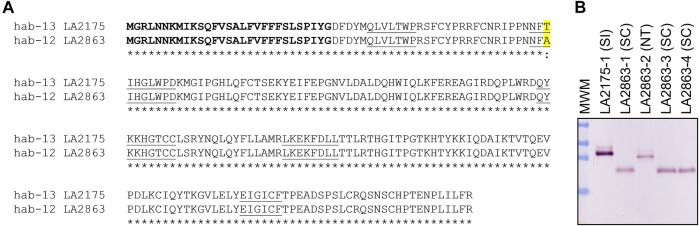
Alignment and immunoblot of hab-12 S-RNase. **(A)** Deduced amino acid sequences of hab-12 (GenBank OK091161) and hab-13 (GenBank OK091162) from SC accession LA2863 and an SI plant from MP accession LA2175, respectively. A shorter but identical hab-13 sequence was identified in MP accession LA1391 (not shown). The predicted signal peptide is bolded, the single amino acid substitution is highlighted in yellow and conserved sequences C1-C5 are underlined. Asterisks indicate conservation between both sequences. **(B)** Immunoblot of stylar extracts using the tSRNC2 antibody raised to the conserved C2 region of S-RNases. MWM, molecular weight marker; SI, self-incompatible; SC, self-compatible; NT, mating system not tested.

The newly discovered *hab-12 S-RNase* allele (Figure 6A) was identified in SC accession LA2863 collected near Macará in southern Ecuador (Figure 4). In accession LA2863, SC plants express an S-RNase protein that appears smaller than normal on immunoblots (Figure 6B). RNA-seq data using RNA from styles of SC plants revealed high expression (33,000–46,000 FPKM) of a single *S-RNase* allele that we named *hab-12*. The nucleotide sequence (including the sequence of the single intron) of the *hab-12* allele was identical to that of the *hab-13* *S-RNase* allele found in SI plants of the mixed SI/SC accession LA2175 and mixed SI/SC accession LA1391 from northern Peru except for a single A→ T transition that creates a missense mutation (Supplementary Figure S13) resulting in a single Thr→ Ala amino acid substitution within the conserved C2 region (Figure 6A). This substitution in the hab-12 protein would eliminate the only potential N-glycosylation site in the protein, a modification which is apparently not required for allele-specific S-RNase function (Karunanandaa et al., 1994; Soulard et al., 2013) but may affect S-RNase uptake, stability or targeting in pollen tubes (Williams et al., 2015). The lack of glycosylation could explain the apparent low molecular weight of the hab-12 S-RNase on immunoblots (Figure 6B). Interestingly, not all LA2863 individuals showed the same pattern on immunoblots (Figure 6B, individual 2), suggesting that *hab-12* is not fixed in this accession.

The SC type found segregating in accession LA2863 was designated as SC group SC-7 (Table 1). Since the reproductive phenotype of the SC-7 group had not been previously characterized, we performed test crosses to assess different types of reproductive barriers in this group (Supplementary Figure S14). We confirmed an SC mating system (self-pollen tubes reach ovaries and fruits are formed in self pollinations), determined that IRBs are intact in SC-7 (pistils reject pollen tubes of cultivar tomato and of *S. neorickii*) and that SC-7 plants do not have defects in pollen resistance factors (SC-7 pollen tubes are accepted by pistils of SI accession LA1777). Further, SC-7 pistils do not reject pollen tubes of accession SC-2 group accession LA0407, indicating that SC-7 pistils lack the inter-population barriers that are found in SI and in SC-4 accessions (Broz et al., 2017). *HT-A* sequences were identified in SC plants in the SC-7 accession LA2863 and appear to be expressed and functional (Supplementary Figure S12).

##### The *LhgSRN-1 S-RNase* Allele

In the most northern accessions of *S. habrochaites* (the SC-2 group), the *LhgSRN-1* *S-RNase* allele is associated with SC (Figure 4). Segregation analysis indicated that the *LhgSRN-1* allele is at, or near, the *S*-locus (Supplementary Table S2). Previous studies showed that although this allele encodes a seemingly functional S-RNase, it is not expressed at the RNA or protein level (Kondo et al., 2002a; Covey et al., 2010; Broz et al., 2017). The SC-2 accessions all possess a Miniature Inverted-repeat Transposable Element (MITE) in the promoter of the *LhgSRN-1* gene (Kondo et al., 2002a; Broz et al., 2017), and this MITE was assumed to be responsible for the lack of expression. However, we identified highly similar, presumably ancestral, functional *LhgSRN-1*-like alleles in SI accessions throughout the species range that harbored the same MITE sequence in their promoter regions (Supplementary Table S5). Analysis of stylar RNA from several of these SI and mixed SI/SC accessions showed high levels of expression of the *LhgSRN-1-*like alleles (Table 2). This indicates that the presence of the MITE is not responsible for the lack of expression of *LhgSRN-1* in SC-2 accessions. Thus, *LhgSRN-1* is classified as a silenced allele (Table 1), but the silencing mechanism is currently unknown.

**TABLE 2:** Expression of silenced/expressed *S-RNase* alleles in SC and SI *S. habrochaites* populations. Transcriptional expression was analyzed by either RT-PCR or RNA-seq analysis using stylar RNA. S-RNase protein was analyzed by immunoblotting with stylar protein extracts. NT = not tested. ^a^This study, ^b^ Covey et al., 2010, ^c^ Broz et al., 2017, ^d^ Broz et al., 2021, ^e^Multiple individuals tested negative, and a single individual tested positive, ^f^This population segregates for *hab-7* and *LhgSRN-1*, and all individuals tested that contained *hab-7* were positive for *hab-7* mRNA with RNA-seq and for S-RNase protein with immunoblotting (Supplementary Figure S15), ^g^Two clones of this plant were grown in either the field or in a growth chamber and gave identical results, ^h^This plant was a genetic sibling of plant (LA2119 x LA2175)-851 and was grown in the greenhouse.

Allele	Plant type/accession	SI/SC	*S-RNase* transcript expression	S-RNase protein
*LhgSRN-1*	LA0407	SC	Negative with RT-PCR^b^	Negative^c^
*LhgSRN-1-like hab-16*	LA2868	SI	NT	Positive^c^
*LhgSRN-1-like hab-17*	LA2099	SI/SC	FPKM = 22,840^a,f^	NT
*LhgSRN-1-like hab-4*	LA1353	SI	Positive with RT-PCR^b^	NT
*LhgSRN-1-like hab-9*	LA0094	SI	Positive with RT-PCR^d^	Positive^d^
*hab-7*	LA2119	SC	FPKM = ∼ 40^a^	Negative^c,e^
*hab-7*	LA2119	SC	NT	Positive^a^
*hab-7*	EC40	SC	NT	Positive^a^
*hab-7*	PI250315	SC	FPKM = 30,000–56,000^a^	Positive^a,f^
*hab-7/hab-15*	(LA2119 x LA2175)-851^g^	SC	FPKM = ∼ 40/35,000^a^	NT
*hab-7/hab-15*	(LA2119 x LA2175)-852^h^	SC	FPKM = 24,000/29,000^a^	NT

**FIGURE 7:**
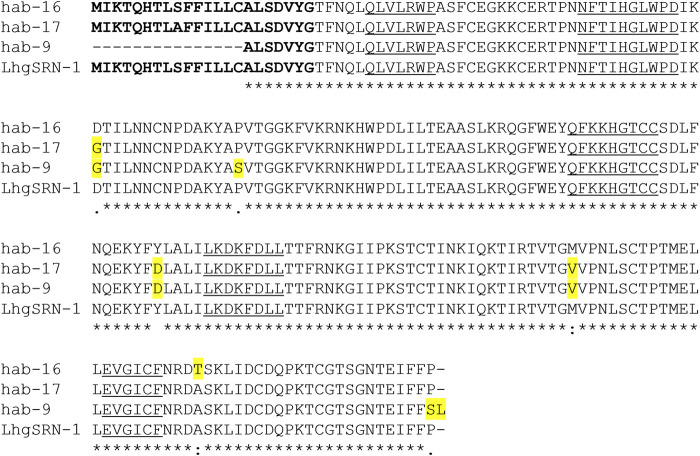
Amino acid sequence alignment of *S. habrochaites* SI-associated LhgSRN-1-like hab-16, hab17 and hab-9 S-RNases with SC-associated LhgSRN-1 S-RNase. Predicted amino acids of LhgSRN-1 S-RNase from SC-2 group accessions are aligned with predicted hab-16 sequences (GenBank OK091165) from SI individuals from SI accession LA2868, hab-17 (GenBank OK091166) from mixed SI/SC LA 2099 (identical sequences were recovered from SI EC7 and EC10 collections as well as mixed SI/SC accession LA1391, not shown) and hab-9 in LA0094, GenBank MW183816 (identical sequences were recovered from accession LA1648). The LhgSRN-1-like hab-4 sequence is not shown due to its relatively short length. The predicted signal peptide is bolded, amino acid substitutions are highlighted in yellow and conserved sequences C1-C5 are underlined. Asterisks indicate conservation between all sequences.

The *LhgSRN-1*-like alleles identified in SI and SI/SC accessions include *hab-4*, *hab-9*, *hab-16* and *hab-17* (Table 2), and their deduced amino acid sequences differ from LhgSRN-1 by between one and six amino acids (Figure 7). The closely related hab-16 sequence has a single Ala/Thr substitution relative to LhgSRN-1, and the SI accession from which this sequence is derived (LA2868) is geographically close to the SC-2 group accessions (Figure 4; Supplementary Table S4). In addition, LA2868 and SC-2 accessions display similar population structure (Landis et al., 2021). Together, the data strongly suggest that *hab-16* is the ancestral functional allele of *LhgSRN-1*.

Pistils of SC-2 accessions show a reduction in strength of IRBs against pollen tubes of both cultivated tomato and SC *S. neorickii* (Broz et al., 2017) compared to their SI counterparts which contain robust IRBs (Supplementary Table S5; Covey et al., 2010; Broz et al., 2017; Broz et al., 2021; Landis et al., 2021). This suggests that loss of S-RNase expression in the SC-2 group diminishes but does not eliminate IRBs. All SC-2 group accessions were found to contain HT-protein, except for a single accession (LA1223) which lacks all IRBs and was designated SC-3 (Broz et al., 2017).

##### The *hab-7 S-RNase* Allele

The recently described *hab-7* allele (Landis et al., 2021) resides in SC-1 accessions (Broz et al., 2017; Landis et al., 2021). SC-1 accessions are generally found in a north-south corridor centering on the town of Loja in southern Ecuador, and in 2014 SC populations with the *hab-7* allele were found to persist in this region, as well as near the town of Cariamanga (EC40) (Figure 4). Previous studies have shown that the SC-1 group possesses S-RNase independent IRBs (Broz et al., 2017), and population structure analysis indicates that the SC-1 group has differentiated from ancestral SI populations and other SC groups (Landis et al., 2021). Segregation analysis indicated that the *hab-7* allele is at, or near, the *S*-locus (Supplementary Table S2).

Although the *hab-7 S-RNase* appears to encode a functional S-RNase, with a single conservative Val/Leu amino acid difference with the *S. peruvianum* S-13 S-RNase (Landis et al., 2021), it is generally not expressed in SC-1 plants (Broz et al., 2017), and the silencing mechanism remains unknown. Unexpectedly, our RNA-seq and immunoblot studies indicate that the normally silenced *hab-7* allele can become activated, resulting in high levels of expression in styles (Table 2). For example, immunoblotting showed that hab-7 S-RNase protein is expressed in plants of the SC population EC40. Further, we found that hab-7 was expressed in a clone of a single LA2119 plant (an SC-1 accession containing *hab-7*) grown in a greenhouse at Colorado State University (Supplementary Figure S15; Table 2), but not in clones of the same plant grown in the field (Table 2), suggesting that environmental conditions may influence expression. Changes in genetic background may also activate *hab-7* expression. For example, accessions from a region of central Ecuador where SC-1/SC-2 hybridization may have occurred (represented by accession PI251305), segregate for both *hab-7* and *LhgSRN-1*. In these accessions, plants containing the *hab-7* allele also express *hab-7* mRNA to high levels in styles, about 1,000x higher than when the gene is silenced (Table 2). Styles of plants heterozygous for *hab-7* exhibit about half of the expression level compared to *hab-7* homozygotes, which is also suggested by immunostaining for S-RNase protein (Supplementary Figure S15). In another example of expression induced by hybridization, when we produced *hab-7/hab-15* hybrids for RNA-seq and segregations studies, we found that some, but not all, plants expressed the *hab-7* allele at a level comparable to that of the functional SI *S-RNase* allele *hab-15* (Table 2). Thus, although *hab-7* is classified as a silenced allele (Table 1), our data suggest that different genetic or environmental conditions can lead to robust transcriptional activation.

##### The *hab-6 S-RNase* Allele

In contrast to the multiple mating system transitions seen at the northern *S. habrochaites* species margin, there has been a single SI → SC transition at the southern species margin in central Peru, producing the SC-4 group, which represents nearly 25% of the species range (Covey et al., 2010; Broz et al., 2021). The *hab-6* allele associated with the SC-4 group is expressed but encodes a low activity protein which does not appear to function in SI. However, the SC-4 group retains robust IRBs (Covey et al., 2010; Broz et al., 2021).

## Discussion

### A Pathway to SC – Loss of Function Mutations and Silencing of *S-RNase* Genes

**FIGURE 8:**
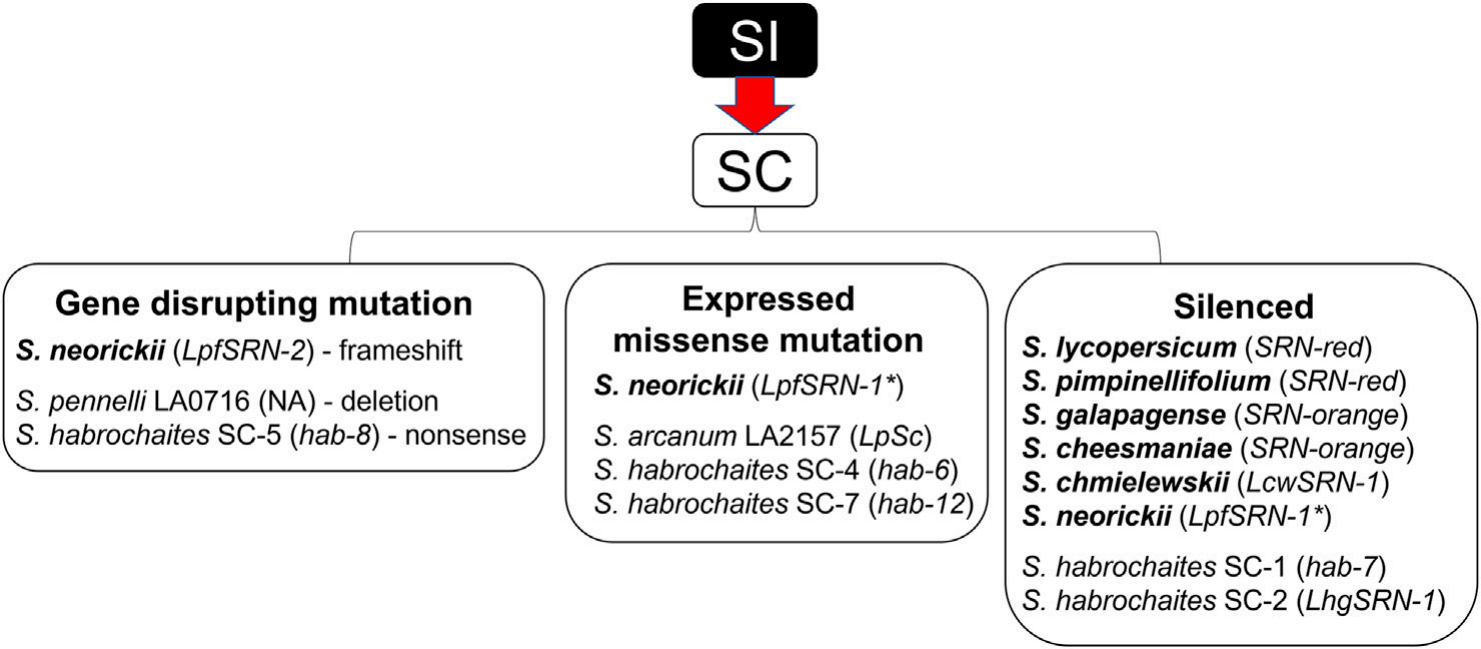
Overview of *S-RNase* alleles associated with self-compatibility (SC) in the tomato clade. Species that are fully SC are shown in bold, for other predominately SI species, SC populations or population groups are listed. Names of *S-RNase* alleles are shown in parentheses. Missense mutations result in low S-RNase activity. *LpfSRN-1 is silenced in some accessions but is expressed and has low activity in others. For additional details see Table 1 and manuscript text.

Although mutations in *S-RNase* genes can drive mating system transitions from SI to SC, the specific nature of these mutations is often not well characterized. Here we examined the structure, origin and expression of 12 *S-RNase* alleles associated with SC species and populations in the tomato clade (Table 1; Figure 8). In three cases, the reason for *S-RNase* gene dysfunction due to mutations is quite clear: one species contains a gene deletion (*S. pennellii* SC accession LA0716), one allele has a frame-shift mutation (*S. neorickii LpfSRN-2*), and another allele contains a nonsense mutation (*S. habrochaites hab-8*). In three other cases*, S-RNase* alleles are expressed but produce proteins that are non-functional in SI (*S. arcanum LpSc*, and *S. habrochaites hab-6* and *hab-12*). Here SC is predicted to result from changes in critical amino acid residues that are likely important for S-RNase protein function. We found that five alleles are transcriptionally silenced (*SRN-red* in *S. lycopersicum* and *S. pimpinellifolium* and *SRN-orange* in *S. galapagense* and *S. cheesmaniae*, *S. chmielewskii LcwSRN-1*, and *S. habrochaites LhgSRN-1* and *hab-7*), but the silencing mechanisms remain unknown. Finally, one *S-RNase* allele (*LpfSRN-1*) can be either transcriptionally silenced or actively transcribed and translated to produce an S-RNase that does not function in SI. In this case, the S-RNase has very low activity (Kondo et al., 2002a), but it appears that the transition to SC is likely to have resulted from a gain-of-function in the male component of the *S*-locus (Markova et al., 2017). Still, although gain-of-function acquisitions of pollen SLFs are expected to be rare given the architecture of SI in the Solanaceae, it should be noted that without complete sequencing of *S*-loci and a better understanding of the interactions between S-RNases and their cognate SLFs, it is impossible to completely rule out pollen-first mutations even when *S-RNase* alleles are non-functional.

In addition to those in the tomato clade, SC populations have been detected in numerous SI species within the Solanaceae (Goldberg et al., 2010; Robertson et al., 2011). However, in most cases, the genes underlying these SC transitions remain unknown. Studies on SC populations of *S. chacoense* (Qin et al., 2001), *S. carolinense* (Mena-Ali and Stephenson, 2007) and *Petunia axillaris* (Tsukamoto et al., 1999; Tsukamoto et al., 2003a), have found low or no expression of specific *S-RNase* alleles, and genetic factors modifying *S-RNase* expression are likely the causative factors leading to SC (Qin et al., 2001; Tsukamoto et al., 2003a). In the Rosaceae and Rutaceae families, which also exhibit gametophytic SI with *S-RNase* and *SLF* alleles at the *S*-locus, there is evidence that SC can result from deletion (Sassa et al., 1997), mutation (Liang et al., 2020) or silencing (Fernandez I Marti et al., 2014) of *S-RNase* genes, or from pollen-side mutations (Vilanova et al., 2006). We believe the current study, combined with previous results, establishes the tomato clade, with its array of SC species and populations, as the premier study system for understanding the molecular basis of S-RNase-based SI to SC transitions. However, continued work in other plant species will be critical to understanding the diverse mechanisms by which SC arises.

### Expression of SC-Associated S-RNases can Alter IRBs

When functional S-RNases are expressed (in conjunction with HT and other pistil SI factors), they can function in both SI and in S-RNase-dependent IRBs (Tovar-Méndez et al., 2014; Tovar-Méndez et al., 2017). When S-RNases are not expressed, IRBs can be entirely absent, as in SC red- and orange-fruited tomato species and SC *S. chmielewskii*, or severely weakened, as in the SC-2 group of *S. habrochaites* (Baek et al., 2015). Alternatively, when S-RNases are not expressed, S-RNase-independent IRBs can be robust, entirely replacing S-RNase-dependent IRBs. Examples include SC *S. pennellii* accession LA0716, in which the *S-RNase* gene is deleted (Covey et al., 2010), and the SC-1, SC-5 and SC-6 groups of *S. habrochaites* (Broz et al., 2017; Landis et al., 2021), in which S-RNases are not expressed but IRBs are intact.

Intriguingly, in cases where a low/no activity S-RNase protein is expressed and plants are SC, IRBs can vary. In the single *S. arcanum* SC accession LA2157, which expresses defective S-RNase LpSc, pistil-side IRBs are severely compromised but still weakly active against pollen tubes of red- and orange-fruited tomato species (Baek et al., 2015). In SC-4 and SC-7 groups of *S. habrochaites*, which express hab-6 and hab-12 S-RNases respectively, self-pollen tubes are not rejected, but interspecific pollen tubes are rejected [(Covey et al., 2010; Broz et al., 2021), Supplementary Figure S14]. In these cases, it is not clear whether the S-RNases that are defective in SI can still function in IRBs, or if S-RNase-independent mechanisms are responsible for rejection of interspecific pollen tubes.

In this study, we found that expression of low-activity LpfSRN-1 S-RNase was required for IRBs in *S. neorickii* populations (Figures 2, 3; Supplementary Figure S6; Supplementary Table S3). However, our results also indicate that another pistil factor(s) is required along with LpfSRN-1 for fully functioning IRBs. We found that HT-proteins, which play a role in both S-RNase-dependent and S-RNase independent IRBs (Tovar-Méndez et al., 2014; Tovar-Méndez et al., 2017) were expressed in all *S. neorickii* accessions (Supplementary Figure S7), suggesting that a different factor is involved. Possibilities include the additional SI and UI factors that have been identified within the Solanaceae (Hancock et al., 2005; Jiménez-Durán et al., 2013; Garcia-Valencia et al., 2017; Qin and Chetelat, 2021). Clearly, more work will be required to identify this additional IRB factor(s).

### Transcriptional Plasticity of *S-RNase* Alleles

Our study uncovered transcriptionally silenced *S-RNase* alleles, and the mechanism(s) underlying the silencing of apparently intact *S-RNase* alleles is currently unknown. Genomic sequencing could clarify whether expression depends on sequence variation of promoters or other regulatory sequences. In the two cases where there are virtually identical pairs of silenced and expressed alleles (*LcwSRN-1* and *LpSc*, *LhgSRN-1* and *hab-16*) direct sequence comparison of regulatory regions should be possible. However, *S-RNase* expression may depend on additional genetic factors, as indicated by our crossing experiments in *S. neorickii* showing heritable variation in *LpfSRN-1* expression. The activation of *hab-7* transcription in hybrids of *S. habrochaites* (*hab-7/hab-15* heterozygotes and SC-1/SC-2 hybrids, Table 2) also points to a genetic basis for transcriptional activation of *S-RNase*. Our results also suggest that there may be environmental influences on *S-RNase* expression, given the apparently spurious reactivation of *hab-7* in a greenhouse-grown versus field-grown clone from the same plant. Previous work indicates that levels of S-RNase can vary between plants of different genetic backgrounds, and even between styles on the same plant, which may be due to differences in factors influencing expression, activity or turnover of S-RNase (Qin et al., 2006; Mena-Ali and Stephenson, 2007; Fernandez I Marti et al., 2014). Mapping studies combined with whole genome sequencing could shed light on the genetic mechanisms underlying silencing, while studies of DNA methylation, chromatin modification and small RNA expression can help clarify if silencing and reactivation result from epigenetic modifications.

It has been widely assumed that the transition from SI to SC is irreversible (Stebbins, 1974; Igić and Busch, 2013), in part because SC is often the result of one or more loss-of-function mutations; and functional reconstitution of these genes would be extremely rare. In this scenario, the genetic diversity normally maintained by outcrossing is lost, putting selfing populations at a higher risk for extinction than their SI counterparts. The plasticity of *S-RNase* expression that we observed suggests that mating system transitions may have the potential for reversibility. This would have important agronomic implications, as a lack of SC germplasm limits many plant breeding programs, and additional tools to manipulate mating system would be extremely valuable (Muñoz-Sanz et al., 2020). In addition, reversible *S-RNase* silencing could influence the evolution and spread of plant populations. Although highly speculative, it is interesting to consider a scenario under which a temporary pause on enforced outcrossing due to transient silencing of an *S-RNase* allele could promote the successful colonization of novel habitats. Plants carrying a single copy of the silenced *S-RNase* would exhibit the SC phenotype, but other *S*-haplotypes would be retained in heterozygous individuals. As long as a sufficient number of diverse *S*-haplotypes (>3) is preserved in a locally adapted founder population, SI systems could theoretically become reactivated with the reversal of *S-RNase* gene silencing, preventing a permanent loss of genetic diversity. Of course, if selfing syndrome characters such as reduced flower size evolve in conjunction with SC, a simple reversal of *S-RNase* expression may not be sufficient to reinstitute SI. Our finding that the transition to SC in *S. habrochaites* is typically not associated with reduced flower size may suggest that SC plants can continue to recruit pollinators, facilitating outcrossing even when selfing is possible. In this case, we would not expect changes in floral morphology to inhibit the putative reversion from SC to SI. A greater understanding of the frequency and mechanisms of *S-RNas*e silencing will determine the potential for this scenario.

## Conclusion

An analysis of SI to SC transitions in the tomato clade reveals a diverse array of mutations that can lead to the loss of *S-RNase* function. This likely represents only a fraction of the diversity that lies within the Solanaceae, and more broadly in S-RNase-based systems of SI. The nature of *S-RNase* mutations can also lead to changes in IRBs, influencing interactions between species. Intriguingly we identified a number of cases in which *S-RNases* can undergo transcriptional silencing, which in some cases can be reversed. Taken together, our results, suggest that *S-RNase* expression, and potentially mating system transitions, may be more dynamic than has previously been thought.

## Data Availability

The datasets presented in this study can be found in online repositories. The names of the repository/repositories and accession number(s) can be found below: https://www.ncbi.nlm.nih.gov/genbank/, OK091159 - OK091166 https://www.ncbi.nlm.nih.gov/genbank/, PRJNA310635.
