# Supplementary material for: S-RNase Alleles Associated With Self-Compatibility in the Tomato Clade: Structure, Origins, and Expression Plasticity
Source: Front Genet. 2021 Dec 6;12:780793. doi: 10.3389/fgene.2021.780793 (PMC8685505; doi:10.3389/fgene.2021.780793)
Supplement: Supplementary file 1 [file DataSheet1.PDF]

Supplemental Material – Broz, Miller, Baek *et al*.

Allele	Primer name	Sequence (5'-3')	Notes
**SC-associated**			
SRN-red and *SRN-orange*	CM63F	ATGAGAACGAACGGTGAAGTAG	5' upstream of ATG start codon
SRN-red and *SRN-orange*	PAB4lycoR	TTATCTAAATAAAATTGCCGTACCG	Includes TAA stop codon
LpfSRN-1	5'neosrn-1-FP7	ATGGTTAAACCACAACTCACAGCA	Includes ATG start codon
LpfSRN-1	3' neosrn-1-RP7	TGTTGTTCAGCGAAAAAATATTTTTCCGG	Includes TGA stop codon
LpfSRN-2	5' neosrn-2-FP-1	GCGAT TAACCCCTTGAGG	5' to premature stop codon
LpfSRN-2	3' neosrn-2-RP-1	CCAATCTCATTAAGTTCCACATTTCC	Within C5 conserved region
LhgSRN-1	AB7F	ATGATTAAAACACAGCACACGTTATC	Includes ATG start codon
LhgSRN-1	AB10R	TTAAGGAAAGAAAATTTCCGTATTTCC	Includes TAA stop codon
hab-6	AB5	GACTTAGCTACCAGACATCC	5' to ATG start codon
hab-6	1927R	CTCACGGAATTGTTCCTCGATC	817bp 3' of start codon
hab-7	CM48F	CTAGAGAATGTATAAGTCACAGATCC	Includes ATG start codon
hab-7	CM49R	ATTCCCTTCCACTGGTTCC	107bp 3' to stop codon
hab-8	CM67F	GATTAAACCACAGCTAATGTCAG	Contains second 5' ATG potential start codon
hab-8	CM70R	TGTCGTTTCAATAATCGAACAGGG	51bp 3'to TGA stop codon (not the premature one)
hab-12	EH1F	TCCAACTAAAATGTTTCAATACCCC	5' to ATG start codon
hab-12	EH2R	GGAATCTTCTTAAACATAGCCATAAGC	3' to stop codon
**SI-associated**			
hab-10	AB32F	CAAGTCCGTAGTACTGAATAACTGC	In C2 region
hab-10	AB33R	TGGCTTTTGTATGCACTTGAGG	Near C5 region
hab-15	AB16F	ACTGCAAAATGTTTAAACCACAACTCAAATC	Includes start codon
hab-15	AB18R	AGGAAAGGAAGTTGTTCAGCTTCG	Includes stop codon
**DNA/RNA Controls**			
Nitrate reductase	4444F	GGATACACAGAAGATTGCTCATG	Solyc01g087550.2
Nitrate reductase	4444R	TCGAACTACAGGCTTCCTAGC	Solyc01g087550.2
CAC, Clathrin Adapter Complex	5'CACFP	CCTCCGTTGTGATGTAACTGG	SGN-U314153
CAC, Clathrin Adapter Complex	3'CACRP	TTGGTGGAAAGTAACATCATCG	SGN-U314153

Supplemental Table 1. *S-RNase* allele-specific and DNA quality/RT-PCR control primers. Note: In our hands, no amplicon was produced using *S. pimpinellifolium* DNA (two different accessions) and several different primer sets.

**Allele(s) tested**	Female allele	Male allele 1 F_1		Male allele 2 F_1	# F_2 w/ Female	# F_2 w/ Male 1	# F_2 w/ Male 2	# F_2 w/ Male 1+2	P-value
LpfSRN-1, LpfSRN-2	*SRN-red*	*LpfSRN-1*	*LpfSRN-2*	*hab-7*	92	32	60	0	
	LhgSRN-1	*LpfSRN-1*	*LpfSRN-2*	*hab-7*	20	9	11	0	
	Total observed				112	41	71	0	
	Total Expected (at S-locus)					56	56	0	0.05879
	Total Expected (not at S-locus)					42	42	28	< 0.00001
LhgSRN-1	*SRN-red*	*LhgSRN-1*	*na*	*hab-10*	106	58	47	1*	
	SRN-red	*LhgSRN-1*	*na*	*hab-6*	30	16	14	0	
	SRN-red	*LhgSRN-1*	*na*	*hab-7*	44	20	24	0	
	Total observed				180	94	85	1	
	Total Expected (at S-locus)					90	90	0	0.67323
	Total Expected (not at S-locus)					68	68	45	< 0.00001
hab-7	*SRN-red*	*hab-7*	*na*	*hab-15*	124	52	70	2*	
	LhgSRN-1	*hab-7*	*na*	*hab-15*	47	23	24	0	
	hab-8	*hab-7*	*na*	*hab-15*	32	20	12	0	
	Total				203	95	106	2	
	Total Expected (at S-locus)					102	102	0	0.40743
	Total Expected (not at S-locus)					76	76	51	< 0.00001

Supplemental Table 2. Segregation analysis of SC-associated *S-RNase* alleles *LpfSRN-1, LpfSRN-2, LhgSRN-1* and *hab-7.* The presence of *S-RNase* alleles in homozygous female and heterozygous male alleles in parents and progeny plants in a segregation study was assessed with allele-specific PCR. *Plants were discarded before they could be re-tested, and likely represent false positives due to sample mixing. *na* = not applicable The Freeman Halton extension of Fishers exact test was used to test the hypothesis that various proposed *S*-alleles are at the *S*-locus, based on the results of progeny segregation. Here, tests were performed for alternative hypotheses: (H1) the allele is at the *S*-locus and we expect that male alleles are never inherited together, (H2) the allele is not at the *S*-locus and we expect male alleles will be inherited together 25% of the time. In every instance the probability (p-value) of H1 exceeds that H2, and in each case for H1 the p-value exceeds 0.05, such that we accept H1 and reject H2.

Plants	Accept pollen tubes, do not express protein	Accept pollen tubes, express protein	Reject pollen tubes, do not express protein	Reject pollen tubes[a], express protein
(D x C) F_1	0	0	0	4
(D x C -1) F_2 plants	8	3	0	3
(D x C -2) F_2 plants	3	3	0	7
(D x C -3) F_2 plants	7	4	0	2
(D x C -4) F_2 plants	2	2	0	5
All F_2 plants (49)	20	12	0	17

Supplemental Table 3. Phenotypes of *S. neorickii* intercrosses between accessions in D and C geographic groups. Group C (LA2047) and D (LA1322) individuals were crossed to generate F1 plants. Four F_1 plants were self-pollinated to generate F_2 plants. *S. lycopersicum* pollen tube growth and expression of S-RNase were assessed in styles of F_1 and F_2 plants. [a]In two plants, pollen tube rejection was late, with pollen tubes traversing the style, but not entering ovaries.

ID	Accession	Mating system	Allele	Lat	Lon
1	LA4656/ ECU1498	SC-2	*LhgSRN-1*	-1.04889	-80.0903
2	LA1624	SC-2	*LhgSRN-1*	-1.3	-80.5833
3	PI129157	SC-2	*LhgSRN-1*	-1.4	-78.45
4	LA1625	SC-2	*LhgSRN-1*	-1.5	-80.5167
5	LA1266	SC-1	*hab-7*	-2.01111	-78.975
6	PI390515	SC-2	*LhgSRN-1*	-2.17656	-78.8173
7	LA0407	SC-2	*LhgSRN-1*	-2.18056	-79.8836
8	LA1264	SC-1	*hab-7*	-2.18333	-79.1
9	LA1223	SC-3	*LhgSRN-1*	-2.19583	-78.8506
10	PI251305	SC-1 /SC-2	*hab-7/LhgSRN-1*	-2.23802	-78.9047
11	PI134417	SC-2	*LhgSRN-1*	-2.2772	-80.1884
12	LA2119	SC-1	*hab-7*	-3.62222	-79.2381
13	LA2868	SI	Multiple, including *LhgSRN-1*-like *hab-16*	-3.75694	-80.0494
14	LA2128	SC-1	*hab-7*	-3.89361	-78.7803
15	LA1252	SC-1	*hab-7*	-4	-79.2167
16	LA2106	SC-1	*hab-7*	-4.2	-79.2167
17	LA2101	SC-5	*hab-8*	-4.33222	-79.5625
19	EC6	SC-5	*hab-8*	-4.3322	-79.5625
20	EC40	SC-1	*hab-7*	-4.3398	-79.5944
18	LA2864/ LA2098	SI/MP	Multiple, including *hab-8*-like *hab-14*	-4.33333	-79.7833
21	LA2099/ EC7/EC10	MP/SI/SI	Multiple, including *LhgSRN-1*-like *hab-17*	-4.35306	-79.8022
22	LA2863	SC-7	*hab-12*	-4.38333	-79.95
23	LA2175	MP	Multiple, including *hab-12*-like *hab-13*	-5.14167	-79.0083
24	LA1391	MP	Multiple, including *hab-12*-like *hab-13* and *LhgSRN-1*-like *hab-17*	-6.028	-79.022
25	LA2314	SI	Multiple, including *hab-8*-like *hab-11*	-6.41667	-77.8667

Supplemental Table 4. Details for Figure 4, SC-associated *S-RNase* alleles and ancestral SI-associated *S-RNase* alleles in *S. habrochaites* at the northern species margin. Unshaded entries are from Ecuador, shaded entries are from Peru.

**Allele name**	**Accession**	**Province or Department, Country**	**Mating system**	**IRBs**	**Presence of MITE (PCR)**	**S-RNase Expressed**
LhgSRN-1	SC-2 group	Manabí/Guayas, Ecuador	SC	weak[a]	Yes	No
hab-16	LA2868	El Oro, Ecuador	SI	robust[b]	Yes	NT
hab-17	EC7	Loja, Ecuador	SI	robust[c]	Yes	Yes
hab-17	EC10	Loja, Ecuador	SI	robust[c]	Yes	Yes
hab-17	LA2099	Loja, Ecuador	MP	robust[a]	Yes	Yes
hab-17	LA1391	Cajamarca, Peru	MP	robust[a]	Yes	NT
hab-4	LA1353	Cajamarca, Peru	SI	robust[d]	NT	Yes
hab-9	LA1648	Lima, Peru	SI	robust[c]	Yes	NT
hab-9	LA0094	Lima, Peru	SI	robust[e]	Yes	Yes

Supplemental Table 5. *LhgSRN-1* and *LhgSRN1-like S-RNase* alleles across the *S. habrochaites* species range. SC, self-compatible; SI, self-incompatible; IRB, Interspecific Reproductive Barrier; weak = rejects pollen tubes *S. lycopersicum*, but not *S. neorickii*; robust = rejects pollen tubes of all SC species. Miniature Inverted-repeat Transposable Element (MITE); Not tested (NT); MP = mixed population SI/SC mating system. [a]Broz et al. 2017, [b]Landis et al. 2021, [c]This study, [d]Covey et al. 2010, [e]Broz et al. 2021.

```
S20     ------------------------------------------------------------
lyc     ATGTTTAAATCACAGCTCATCACGGCTCTTTTCATATTGTTCTTTTGTCTTTCTCCTATT
pim     ATGTTTAAATCACAGCTCATCACGGCTCTTTTCATATTGTTCTTTTGTCTTTCTCCTATT
gal     ATGTTTAAATCACAGCTCATCACGGCTCTTTTCATATTGTTCTTTTGTCTTTCTCCTATT
che     ATGTTTAAATCACAGCTCATCACGGCTCTTTTCATATTGTTCTTTTGTCTTTCTCCTATT

S20     ------------------------------------------------------------
lyc     TACGGGGATTTTGATTACATGCAACTCGTTTTAACTTGGCCACCATCCTTTTGCTATCCA
pim     TACGGGGATTTTGATTACATGCAACTCGTTTTAACTTGGCCACCATCCTTTTGCTATCCA
gal     TACGGGGATTTTGATTACATGCAACTCGTTTTAACTTGGCCACCATCCTTTTGCTATCCA
che     TACGGGGATTTTGATTACATGCAACTCGTTTTAACTTGGCCACCATCCTTTTGCTATCCA

S20     ---------------AGAACATCGAACAATTTCACGATTCACGGTCTTTGGCCCGAGAAG
lyc     AGGGGTACTTGCAAGCGAACATCGAACAATTTCATGATTCACGGTCTTTGGCCCGAGAAG
pim     AGGGGTACTTGCAAGCGAACATCGAACAATTTCATGATTCACGGTCTTTGGCCCGAGAAG
gal     AGGGGTACTTGCAAGCGAACATCGAACAATTTCACGATTCACGGTCTTTGGCCCGAGAAG
che     AGGGGTACTTGCAAGCGAACATCGAACAATTTCACGATTCACGGTCTTTGGCCCGAGAAG
                        ****************** *************************

S20     AAGGGGTTTCGTCTGGAGTTCTGCTCCGGCGGTAAAGCGTATAAGAAATTTGAACTACAA
lyc     AAGGGGTTTCGTCTGGAGTTCTGCTCCGGCGGTAAAGCCTATAAGAAATTTGAACTACAT
pim     AAGGGGTTTCGTCTGGAGTTCTGCTCCGGCGGTAAAGCCTATAAGAAATTTGAACTACAT
gal     AAGGGGTTTCGTCTGGAGTTCTGCTCCGGCGGTAAAGCCTATAAGAAATTTGAACTACAT
che     AAGGGGTTTCGTCTGGAGTTCTGCTCCGGCGGTAAAGCCTATAAGAAATTTGAACTACAT
        ************************************** ********************

S20     ------------------------------------------------------------
lyc     GTACAACAAATTTTTTTCATAGAATTCTAAATTTTATATTTGATCTCAATTATACTTTTC
pim     ------------------------------------------------------------
gal     GTACAACAAATTTTTTTCATAGAATTCTAAATTTTATATTTGATCTCAATTATACTTTTC
che     GTACAACAAATTTTTTTCATAGAATTCTAAATTTTATATTTGATCTCAATTATACTTTTC

S20     -------------------------------------GATCATATAGTCAATGATCTGGA
lyc     AATTCTATTTATGATAGTCGTTTGCTAATTTTTGCAGGATCATATAGTCAATGATCTGGA
pim     -------------------------------------GATCATATAGTCAATGATCTGGA
gal     AATTCTATTCATGATAGTCGTTTGCTAATTTTTACAGGATCATATAGTCAATGATCTGGA
che     AATTCTATTCATGATAGTCGTTTGCTAATTTTTACAGGATCATATAGTCAATGATCTGGA
                                             ***********************

S20     TCACCATTGGATTCAAATGAAGTTCACCGAACAAGATGCTAAACAAAAACAACCTCTCTG
lyc     TCACCATTGGATTAAAATGAAGTTCACCGAACAAGATGCTAAACAAAAACAACCTCTCTG
pim     TCACCATTGGATTAAAATGAAGTTCACCGAACAAGATGCTAAACAAAAACAACCTCTCTG
gal     TCACCATTGGATTAAAATGAAGTTCACCGAACAAGATGCTAAACAAAAACAACCTCTCTG
che     TCACCATTGGATTAAAATGAAGTTCACCGAACAAGATGCTAAACAAAAACAACCTCTCTG
        ************* **********************************************

S20     GAACCACGAATACACAAGACATGGAAGGTGTTGTTTCAATCTCTACGATCAGAACGCATA
lyc     GAACCACGAATACACAAGACATGGAAGGTGTTGTTTCAATCTCTACGATCAGAACGCATA
pim     GAACCACGAATACACAAGACATGGAAGGTGTTGTTTCAATCTCTACGATCAGAACGCATA
gal     GAACCACGAATACACAAGACATGGAAGGTGTTGTTTCAATCTCTACGATCAGAACGCATA
che     GAACCACGAATACACAAGACATGGAAGGTGTTGTTTCAATCTCTACGATCAGAACGCATA
        ************************************************************

S20     TTTTTTACTAGCCATGCGCTTAAAAGATAAATTAGATCTTGTAAGAACTCTCAGAAATCA
```

```
lyc              TTTTTTACTAGCCATGCGCTTAAAAGATAAATTAGATCTTGTAAGAACTCTCAGAAATCA
pim              TTTTTTACTAGCCATGCGCTTAAAAGATAAATTAGATCTTGTAAGAACTCTCAGAAATCA
gal              TTTTTTACTAGCCATGCGCTTAAAAGATAAATTAGATCTTGTAAGAACTCTTAGAAATCA
che              TTTTTTACTAGCCATGCGCTTAAAAGATAAATTAGATCTTGTAAGAACTCTCAGAAATCA
                 *************************************************** ********

S20              TGGAATTACCCCAGGGACAAAGCATACATTTGATGAAATCAAAAGTGCTATTAAGACCGT
lyc              TAGAATTACCCCAGGGACAAAGCATACATTTGATGAAATCAAAAGTGCTATTAAGACCGT
pim              TAGAATTACCCCAGGGACAAAGCATACATTTGATGAAATCAAAAGTGCTATTAAGACCGT
gal              TAGAATTACCCCAGGGACAAAGCATACATTTGATGAAATCAAAAGTGCTATTAAGACCGT
che              TAGAATTACCCCAGGGACAAAGCATACATTTGATGAAATCAAAAGTGCTATTAAGACCGT
                 * **********************************************************

S20              TATTAATCAAGTAGATCCTGATCTCAAGTGCGTC--------------------------
lyc              TACTAATCAAGTAGATCCTGATATCAAGTGCGTCAAGCATATAAATGGAGTAGAGGAATT
pim              TACTAATCAAGTAGATCCTGATATCAAGTGCGTCAAGCATATAAATGGAGTAGAGGAATT
gal              TACTAATCAAGTAGATCCTGATATCAAGTGCGTCAAGCATACAAATGGAGTAGAGGAATT
che              TACTAATCAAGTAGATCCTGATATCAAGTGCGTCAAGCATACAAATGGAGTAGAGGAATT
                 ** ******************* ***********

S20              ------------------------------------------------------------
lyc              AAATGAGATAGGCATATGTTATACCCCCTCGGCCGGTAGTTTTTATCCATGTCGTCAAAG
pim              AAATGAGATAGGCATATGTTATACCCCCTCGGCCGGTAGTTTTTATCCATGTCGTCAAAG
gal              AAATGAGATAGGCATATGTTATACCCCCTCGGCCGGTAGTTTTTATCCATGTCGTCAGAG
che              AAATGAGATAGGCATATGTTATACCCCCTCGGCCGGTAGTTTTTATCCATGTCGTCAGAG

S20              ------------------------------------------------------------
lyc              TAATACATGCGATGAAACGGGTACGGCAATTTTATTTAGATAATGAATGACTTCCAATGA
pim              TAATACATGCGATGAAACGGGTACGGCAATTTTATTTAGATAA-----------------
gal              TAATACATGCGATGAAACGGG---------------------------------------
che              TAATACATGCGATGAAACGGG---------------------------------------
```

Supplemental Figure 1. Alignment of nucleotide sequences encoding S-RNases in four SC red/orange-fruited tomato species with nucleotides encoding a known functional *S. chilense* S-RNase. S20 = partial coding sequences of *S. chilense* S20 allele, partial codons (GenBank EF680098), lyc = *S. lycopersicum* Heinz 1706 genomic sequence from Sol Genomics Network (https://solgenomics.net) Solyc01g055200.1, pim = *S. pimpinellifolium* LA1589 cDNA coding sequence only from Sol Genomics Network Sopim01g055200.0.1, gal = *S. galapagense* LA0317 genomic PCR product (GenBank OK091157), this study, che = *S. cheesmaniae* LA0522, genomic PCR product, this study (GenBank OK091158). Start codons are highlighted in green, intron sequences for genomic sequences are highlighted in turquoise, and stop codons are highlighted in red. Asterisks indicate conservation between all sequences.

```
S. chi S11        ----------------------------------------------PIPNNFTIHGLWPD
S. chm LcwSRN-1  MFKSQLTSVFFMFLFALSPIYGYFELLELVSTWPATYCYAYGCSRRPIPNNFTIHGLWPD
S. arc LpSc      -----LTSVFFMFLFALSPIYGYFELLELVSTWPATYCYAYGCSRRPIPNNFTINGLWPD
                                                              ********:*****

S. chi  S11       NKSVILNNCNFAKKEDRYTKITDPKKKSELDKRWPQLRYEKLYGIEKQDLWEKEFLKHGS
S. chm LcwSRN-1  NKSVILNNCNFAKKEDRYTKITDPKKKSELDKRWPQLRYEKLYGIEKQDLWEKEFLKHGS
S. arc LpSc      NKSVILNNCNFAKKEDRYTKITDPKKKSELDKRWPQLRYEKLYGIEKQDLWEKEFLKHGS
                 ************************************************************

S. chi S11        CSINRYKQEAYFDLAMKIKDRFDLLGTLRNQGIIPGSTYELDDIERAVKTVSIEVPSLKC
S. chm LcwSRN-1  CSINRYKQEAYFDLAMKIKDRFDLLGTLRNQGIIPGSTYELDDIERAVKTVSIEVPSLKC
S. arc LpSc      CSINRYKQEAYFDLAMKIKDRFDLLGTLRNQGIIPGSTYELDDIERAVKTVSIEVPSLKC
                 ************************************************************

S. chi S11        I------------------------------------------
S. chm LcwSRN-1  IQKPLGNVELNEIGICLDPEAKYTVPCPRIGSCHKMGHKIKFR
S. arc LpSc      IQKPLGNVELNEIGICLDPEAKYTVPCPRIGSCHKMGHKIKFR
                 *
```

Supplemental Figure 2. Amino acid sequence alignment of SC-associated S-RNases from *S. chmielewskii* and *S. arcanum* with a known functional *S. chilense* S-RNase. S. chi S11 = functional S-RNase S11 from SI *S. chilense* (partial codons), GenBank EF680110, S. chim LcwSRN-1 = predicted S-RNase sequence found in SC *S. chmielewskii*, GenBank AB072477, and S. arc LpSc = expressed but non-functional S-RNase from SC *S. arcanum* accession LA2157, GenBank Z26581. The predicted signal peptide is bolded, the single amino acid substitution in LpSc is highlighted in yellow, and conserved sequences C1-C5 are underlined. Asterisks indicate conservation between all sequences.

```
S. arcanum LA2163 S6
att cac ggg ctt tgg ccg gat aac agg tcc aca att ctg cat gac tgc gat gta ccc cct gag …
 I   H   G   L   W   P   D   N   R   S   T   I   L   H   D   C   D   V   P   P   E
LpfSRN-2
att cac ggg ctt tgg ccg gat aac agg tcc aca att ctg cat gac tgc gat gta acc cct tga
 I   H   G   L   W   P   D   N   R   S   T   I   L   H   D   C   D   V   T   P   -
```

Supplemental Figure S3. Alignment of partial codons and amino acids of *SC S. neorickii LpfSRN-2* with SI *S. arcanum* functional *S-RNase* allele *S6*. Partial codons and encoded amino acid sequences in the functional *S6 S-RNase* allele from SI *S. arcanum* accession LA2163, GenBank Z26583.1, are aligned with the same region containing an insertion mutation in SC *S. neorickii LpfSRN-2 S-RNase* allele, GenBank AB072476.1. Inserted A is highlighted in yellow, and premature stop codon in *LpfSRN-2* is highlighted in red.

```
S. peru SP2      -----------------------------------------------------------K
S. neo LpfSRN-1  MVKPQLTAALFIVLFAISPAYGDFDSLQLVLTWPASFCHMNDCVRIAPKNFTIHGLWPDK
                                                                            *

S. peru SP2      EGTVLQKCKPKPNYSNFKEKMFNDLDKHWIQLKYDEYYGEKEQPLWFYQYLKHGSCCQKM
S. neo LpfSRN-1  EGTVLQNCKPKPNYSNFKEKMFNDLDKHWIQLKYDEDYGEKEQPLWFYQYLKHGSCCQKM
                 ******:***************************** ***********************

S. peru SP2      YNQNTYFSLALRLKDKFDLLRTLQIHKIFPGSSYTFKEIFDAIKTATQMDPDLKCTKGAP
S. neo LpfSRN-1  YNQNTYFSLALRLKDKFDLLRTLQTHKIFPGSSYTFKEIFDAVKTATQMDPDLKCTKGAP
                 ************************ *****************:*****************

S. peru SP2      ----------------------------------
S. neo LpfSRN-1  ELYEIGICFTPKADALIPCRQSNTCARTGKIFFR
```

Supplemental Figure 4. Amino acid alignment of functional *S. peruvianum* S-RNase SP2 with *S. neorickii* LpfSRN-1 S-RNase. S. peru SP2 = *S. peruvianum* SP2 S-RNase, GenBank HM357216.1 (partial sequence), S. neo LpfSRN-1 = SC *S. neorickii* LA1322 LpfSRN-1, GenBank AB072475.1. The predicted signal peptide is bolded, amino acid substitutions are highlighted in yellow and conserved sequences C1-C5 are underlined. Asterisks indicate conservation between all sequences.

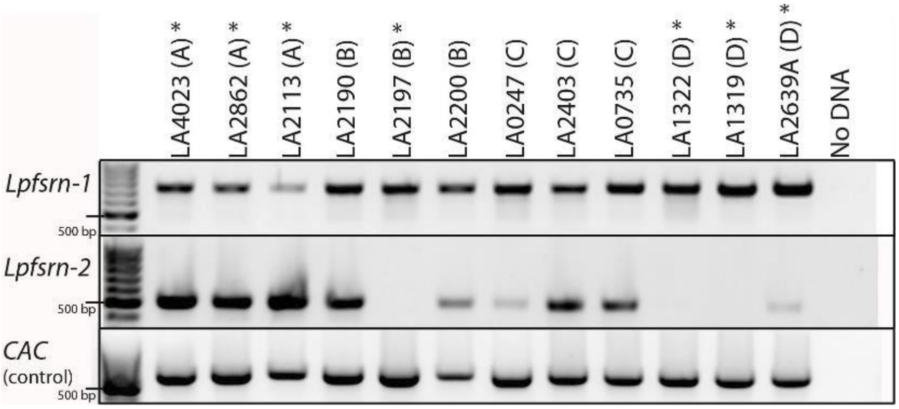

Supplemental Figure 5. Presence of *LpfSRN-1* and *LpfSRN-2 S-RNase* alleles in genomic DNA of *S. neorickii* accessions from different geographic groups. Amplification of *LpfSRN-1* is shown in the top panel and *Lpfsrn-2* is shown in the middle panel. The bottom panel shows amplification of control gene *CAC* (Clathrin Adaptor Complex medium subunit). * = accessions in which pollen tubes from red-fruited species are rejected (IRBs are present).

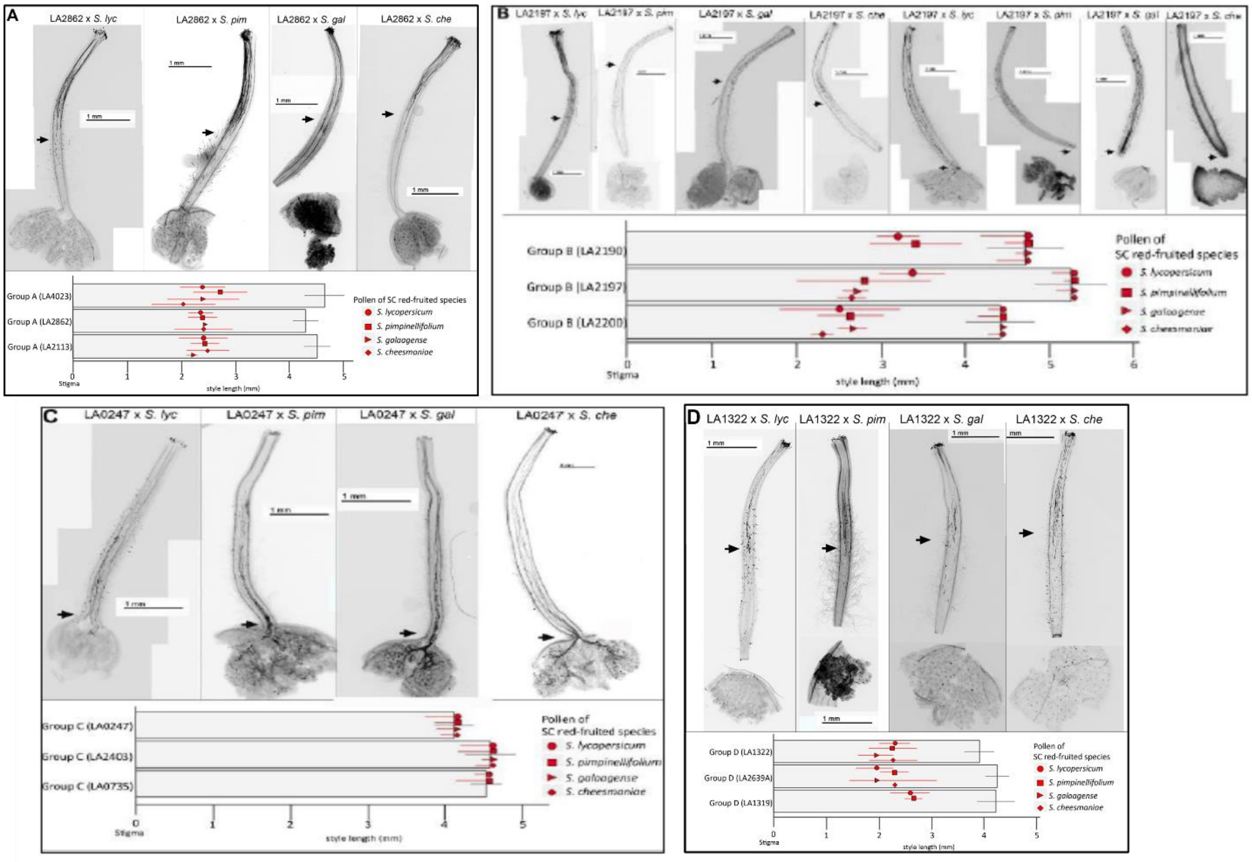

Supplemental Figure 6. Red-fruited tomato species pollen tube growth in styles of different *S. neorickii* geographic group accessions. The top panel of each box shows representative images of crosses for each geographic group of *S. neorickii* with pollen of the four red-fruited species. *S. neorickii* LA2862 is in group A (top left), LA2197 is in group B (top right), LA0247 is in group C (bottom left), and LA1322 is in group D (bottom right). Arrow indicates where majority of pollen tubes stop. Bar = 1mm. The bottom panel of each box shows the length of pollen tubes from red-fruited species in the pistils of three accessions from each group of *S. neorickii*. Rectangles represent style length of *S. neorickii* in mm and the average of majority pollen tube growth (mm) is indicated by red symbols with standard deviation (red bars).

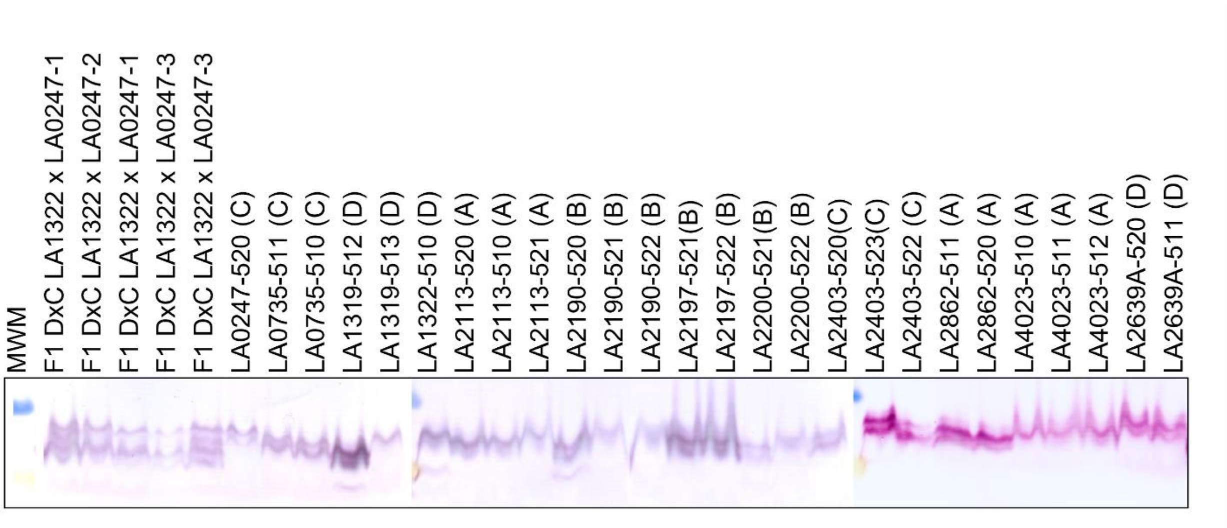

Supplemental Figure 7. Expression of HT-protein in *S. neorickii* styles. Stylar extracts were blotted and probed with an antibody that binds to both HT-A and HT-B proteins.

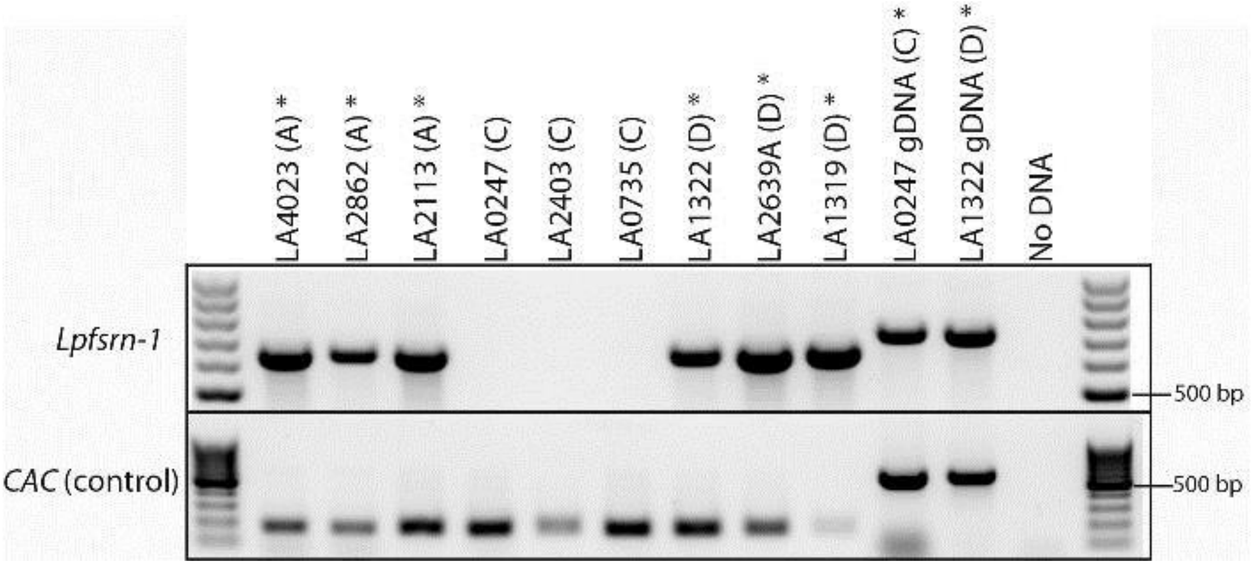

Supplemental Figure 8. Transcriptional expression of *LpfSRN-1* in *S. neorickii* styles. RT-PCR was conducted using stylar RNA from the A, C and D geographic groups of *S. neorickii*. * = accessions in which pollen tubes from red-fruited species are rejected in styles.

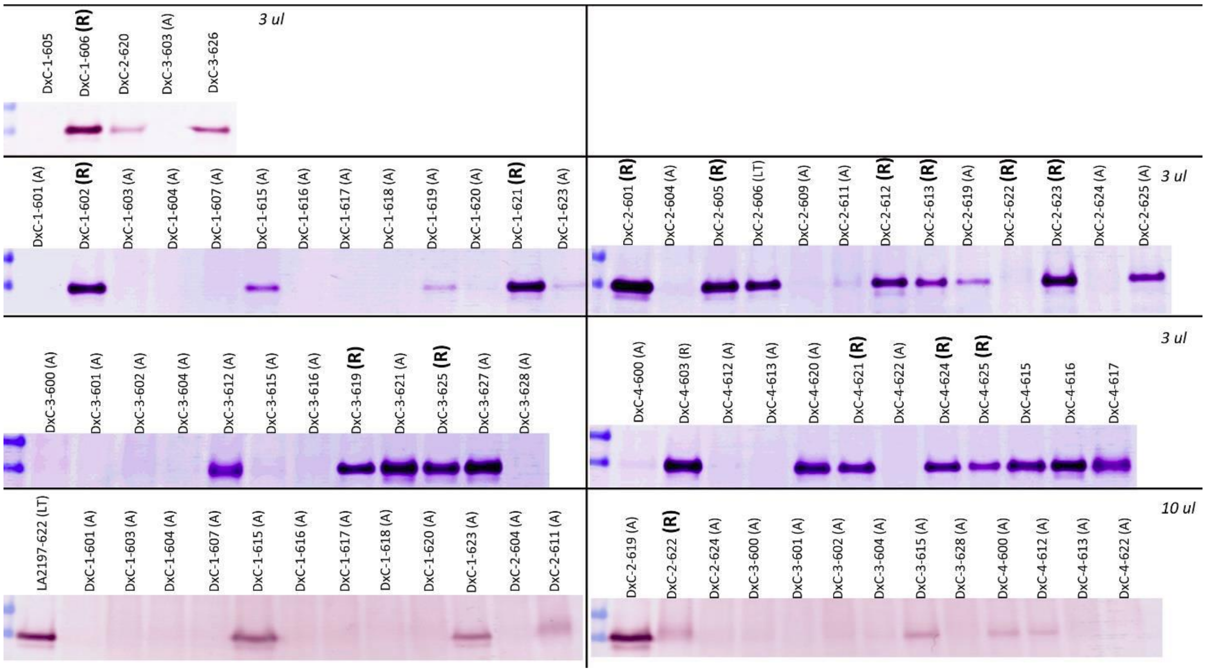

Supplemental Figure 9. Immunoblots and IRB phenotypes of *S. neorickii* geographic group D x geographic group C F_2 plants. Stylar extracts of individual F2 plants were blotted and probed with the general S-RNase antibody raised to the conserved C2 region. 1-4 indicates the different F1 plants that were self-pollinated to generate F_2 plants. The top three panels show immunoblots using 3μl of stylar extract and the bottom panel shows repeated blots using 10μl of extract for samples that exhibited ambiguous results with the smaller volume.

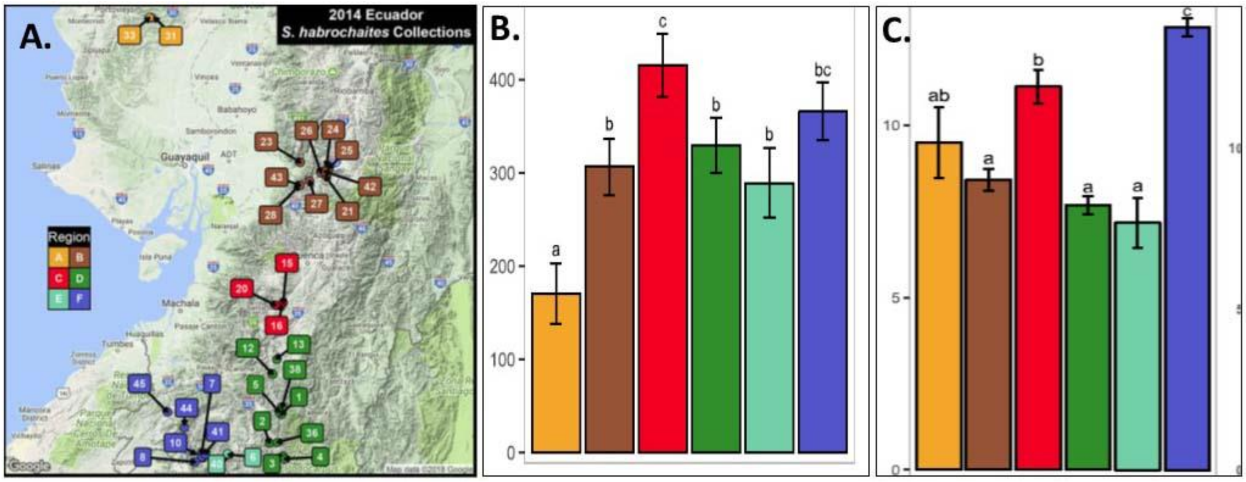

Supplemental Figure 10. Floral characters of SC and SI populations of *S. habrochaites* in Ecuador. A. Locations of SI and SC *S. habrochaites* populations in Ecuador identified in 2014. All populations are SC except for those in blue, which are SI. **B.** Corolla area of flowers of each population, grown in a common garden. **C.** Numbers of floral buds per inflorescence of each population (results were identical *in situ* and in a common garden). Statistically significant groups are denoted with lowercase letters. Shared letters indicate non-significant relationships.

```
LA2098g hab-14   -------------------------CAGCTAATGTCAGTTTTGTTTCTATTTCTTTTTGT
LA2314c hab-11   GTTCGAAAGAGAAATGACTAAACCACAGCTAATGTCAGTTTTGTTTCTATTTCTTTTTGT
LA2101 hab-8     -------------------------CAGCTAATGTCAGTTTTGTTTCTATTTCTTTTTGT
EC6 hab-8        ------------------------------------------------------------

LA2098g hab-14   TTTTTCTCCCGTTTGTGGGGACTTCGATTACTTACAACTTGTTTTACAATGGCCAAGATC
LA2314 hab-11    TTTTTCTCCCGTTTGTGGGGACTTCGATTACTTACAACTTGTTTTACAATGGCCAAGATC
LA2101 hab-8     TTTTTCTCCCGTTTGTGGGGACTTCGATTACTTACAACTTGTTTTACAATGGCCAAGATC
EC6 hab-8        -----CTCCCGTTTGTGGGGACTTCGATTACTTACAACTTGTTTTACAATGGCCAAGATC
                      *******************************************************

LA2098g hab-14   TTTTTGCAAATCAAGATATTGCCCAAATCCAATTCCAAGGAATTTCACGATCCATGGGCT
LA2314c hab-11   TTTTTGCAAATCAAGATATTGCCCAAATCCAATTCCAAGAAACTTCACGATCCATGGGCT
LA2101 hab-8     TTTTTGCAAATCAAGATATTGCCCAAATCCAATTCCAAGGAATTTCACGATCCATGGGCT
EC6 hab-8        TTTTTGCAAATCAAGATATTGCCCAAATCCAATTCCAAGGAATTTCACGATCCATGGGCT
                 *************************************** ** *****************

LA2098g hab-14   ATGGCCTGATAAACAGAGAATTATGCCGATTAACTGCCCGGCCAAAGAGAGCTACAAAAG
LA2314c hab-11   ATGGCCTGATAAACAGAGAATTATGCCGATTAACTGCCCGGCCAAAGAGAGCTACAAAAG
LA2101 hab-8     ATGGCCTGATAAACAGAGAATTATGCCGATTAACTGCCCGGCCAAAGAGAGCTACAAAAG
EC6 hab-8        ATGGCCTGATAAACAGAGAATTATGCCGATTAACTGCCCGGCCAAAGAGAGCTACAAAAG
                 ************************************************************

LA2098g hab-14   TATTACGGTAAACCATATTACTTTTTATTTTTCACCATTGTTTTCTTTCCGTTCTTGCCA
LA2314c hab-11   TATTACG-----------------------------------------------------
LA2101 hab-8     TATTACGGTAAACCATATTACTTTTTATTTTTCACCATTGTTTTCTTTCCGTTCTTGCCA
EC6 hab-8        TATTACGGTAAACCATATTACTTTTTATTTTTCACCATTGTTTTCTTTCCGTTCTTGCCA
                 *******

LA2098g hab-14   TTTTAACTAATTAAATTATAATCCAAGCGTTTACAGGACTTTAAGAAAATCAAACTACTG
LA2314c hab-11   ------------------------------------GACTCTAAGAAAATCAAACTACTG
LA2101 hab-8     TTTTAACTAATTAAATTATAATCCAAGCGTTTACAGGACTTTAAGAAAATCAAACTACTG
EC6 hab-8        TTTTAACTAATTAAATTATAATCCAAGCGTTTACAGGACTTTAAGAAAATCAAACTACTG
                                                     **** *******************

LA2098g hab-14   GAGCAACACTGGCCCGATTTGACCTCCAATCAAGGCAGTGCAGAATTCTGGAGATATCAA
LA2314c hab-11   GAGCAACACTGGCCCGATTTGACCTCCAATCAAGGCAGTGCAGAATTCTGGAGATATCAA
LA2101 hab-8     GAGCAACACTGGCCCGATTTGACCTCCAATCAAGGCAGTGCAGAATTCTAGAGATATCAA
EC6 hab-8        GAGCAACGATGGCCCGATTTGACCTCCAATCAAGGCAGTGCAGAATTCTAGAGATATCAA
                 *******  **************************************** **********

LA2098g hab-14   TACAAGAAACACGGAACGTGTAGTGTGGACCTATATAATCAAGAACAATATTTTGATTTA
LA2314c hab-11   TACAAGAAACACGGAACGTGTAGTGTGGACCTATATAATCAAGAACAATATTTTGATTTA
LA2101 hab-8     TACAAGAAACACGGAACGTGTAGTGTGGACCTATATAATCAAGAACAATATTTTGATTTA
EC6 hab-8        TACAAGAAACACGGAACGTGTAGTGTGGACCTATATAATCAAGAACAATATTTTGATTTA
                 ************************************************************

LA2098g hab-14   GCCATTGAATTAAAAGAGAAGTTTGATCTTTTGAAAACTCTCAAAAATCACGGAATTACT
LA2314c hab-11   GCCATTGAATTAAAAGAGAAGTTTGATCTTTTGAAAACTCTCAAAAATCACGGAATTACT
LA2101 hab-8     GCCATTGAATTAAAAGAGAAGTTTGATCTTTTGAAAACTCTCAAAAATCACGGAATTACT
EC6 hab-8        GCCATTGAATTAAAAGAGAAGTTTGATCTTTTGAAAACTCTCAAAAATCACGGAATTACT
                 ************************************************************

LA2098g hab-14   CCATCAAAAACTAATACAGTTATAGATGTCGAAAAAGCCATCAAGGCCGTAACAAAAGAG
LA2314c hab-11   CCATCAAAAACTAATACAGTTATAGATGTCGAAAAAGCCATCAAGGCCGTAACAAAAGAG
LA2101 hab-8     CCATCAAAAACTAATACAGTTATAGATGTCGAAAAAGCCATCAAGGCCGTAACAAAAGAG
```

```
EC6 hab-8         CCATCAAAAACTAATACAGTTATAGATGTCGAAAAAGCCATCAAGGCCGTAACAAAAGAG
                  ************************************************************

LA2098g hab-14    GTTCCTAACCTCAACTGCATTGGTGACTCTAGTCAAACCATGGAACTACTGGAGATAGGC
LA2314c hab-11    GTTCCTAACCTCAACTGCATTGGTGACTCTAGTCAAACCATGGAACTACTGGAGATAGGC
LA2101 hab-8      GTTCCTAACCTCAACTGCATTGGTGACTCTAGTCAAACCATGGAACTACTGGAGATAGGC
EC6 hab-8         GTTCCTAACCTCAACTGCATTGGTGACTCTAGTCAAACCATGGAACTACTGGAGATAGGC
                  ************************************************************

LA2098g hab-14    ATATGTTTTAACCGAGAGGGAACTACAGTGATTGCTTGTCGTCGACGTTGGAATAATCAT
LA2314c hab-11    ATATGTTTTAACCGAGAGGGAACTACGGTGATTGCTTGTCGTCGACGTTGGAATAATCAT
LA2101 hab-8      ATATGTTTTAACCGAGAGGGAACTACAGTGATTGCTTGTCGTCGACGTTGGAATAATCAT
EC6 hab-8         ATATGTTTTAACCGAGAGGGAACTACAGTGATTGCTTGTCGTCGACGTTGGAATAATCAT
                  ************************** *********************************

LA2098g hab-14    CCTAACGGGAACCAGAAGATTACTCTTCCACCATGAATAATAATACACTTCTTACTT
LA2314c hab-11    CCTAACGGGAACCAGAAGATTACTCTTCCACCATGAATAATAATACACTTCTTACTT
LA2101 hab-8      CCTAACGGGAACCAGAAGATTACTCTTCCACCATGAATAATAATACACTTCTTACTT
EC6 hab-8         CCTAACGGGAACCAGAAGATTACTCTTCCACCATGAATAATAATACACTTCTTACTT
                  *********************************************************
```

Supplementary Figure 11. Nucleotide sequence alignment of *S. habrochaites* SC-associated *hab-8* S-*RNase* allele with *S. habrochaites* SI-associated *hab-14 S-RNase* allele. Genomic *hab-8* allele nucleotide sequences in SC accessions LA2101 and EC6 (partial sequence, GenBank OK091160) are aligned with *hab-14* sequences from SI individuals in accession LA2098 (GenBank OK091163) and *hab-11* sequences from LA2314 (c= cDNA, g = genomic). A sequence identical to that found in LA2098 was found in SI accession LA2864 (not shown). Potential start codons are highlighted in green, single nucleotide substitution is highlighted in yellow, introns are highlighted in turquoise and both the premature stop codon in *hab-8* and normal stop codons in *hab-11* and *hab-14* are highlighted in red. Asterisks indicate conservation between all sequences.

```
HTA SC-1         MAFKANILLIFSLVFMVISSEVIAREMVEANQVQNTFELNNPTLQKKGGGSLFPNIACLG
HTA SC-5         MAFKANILLIFSLVFMVISSEVIAREMVEANQVQNSFELNNPTLQKKGGGSLFPNIACLG
HTA SC-7         MAFKANILLIFSLVFMVISSEVIAREMVEANQVQNTFELNNPTLQKKGGGSLFPNIACLG
                 **********************************:*************************

HTA SC-1         CSCPKKDNKNNNNNNNNDDDDDDDDSFIGNVCKAMCC
HTA SC-5         CSCPKKDNKNNNNNNNNDDDDDDDDSFIGNVCKAMCC
HTA SC-7         CSCPKKDNKNNNNNNNNDDDDDDDDSFIGNVCKAMCC
                 *************************************
```

Supplemental Figure 12. Predicted HT-A amino acid sequences in SC group accessions. Predicted HT-A sequences in SC-1 group accession LA2119 (*hab-7 S-RNase* allele), in SC-5 group accession LA2101 (*hab-8 S-RNase* allele) and in SC-7 group accession LA2863 (*hab-12 S-RNase* allele). Note that the predicted signal peptide is bolded. HT-A sequences for SC-2 and SC-4 groups are in Covey et al., 2010, and HT-A sequences for SC-3 and SC-6 groups are in Landis et al., 2021. Asterisks indicate conservation between all sequences.

```
hab-13c LA1391 ------------------------------------------------------------
hab-12c LA2863 AAACATCTATATATAGAACAAGATGAAAATTTGAGAATGGGTCGTTTGAACAACAAAATG
hab-13g LA2175 -----TCTATATATAGAACAAGATGAAAATTTGAGAATGGGTCGTTTGAACAACAAAATG
hab-12g LA2863 -----TCTATATATAGAACAAGATGAAAATTTGAGAATGGGTCGTTTGAACAACAAAATG

hab-13c LA1391  ------TCACAGTTTGTGTCAGCACTTTTCGTTTTCTTTTTTTCTCTTTCTCCCATTTAT
hab-12c LA2863  ATTAAATCACAGTTTGTGTCAGCACTTTTCGTTTTCTTTTTTTCTCTTTCTCCCATTTAT
hab-13g LA2175  ATTAAATCACAGTTTGTGTCAGCACTTTTCGTTTTCTTTTTTTCTCTTTCTCCCATTTAT
hab-12g LA2863  ATTAAATCACAGTTTGTGTCAGCACTTTTCGTTTTCTTTTTTTCTCTTTCTCCCATTTAT
                      ******************************************************

hab-13c LA1391  GGAGATTTCGACTATATGCAACTTGTCTTAACTTGGCCAAGATCTTTTTGTTACCCTAGA
hab-12c LA2863  GGAGATTTCGACTATATGCAACTTGTCTTAACTTGGCCAAGATCTTTTTGTTACCCTAGA
hab-13g LA2175  GGAGATTTCGACTATATGCAACTTGTCTTAACTTGGCCAAGATCTTTTTGTTACCCTAGA
hab-12g LA2863  GGAGATTTCGACTATATGCAACTTGTCTTAACTTGGCCAAGATCTTTTTGTTACCCTAGA
                ************************************************************

hab-13c LA1391  AGATTCTGCAACAGAATACCTCCGAACAACTTCACGATTCATGGGCTTTGGCCGGATAAG
hab-12c LA2863  AGATTCTGCAACAGAATACCTCCGAACAACTTCGCGATTCATGGGCTTTGGCCGGATAAG
hab-13g LA2175  AGATTCTGCAACAGAATACCTCCGAACAACTTCACGATTCATGGGCTTTGGCCGGATAAG
hab-12g LA2863  AGATTCTGCAACAGAATACCTCCGAACAACTTCGCGATTCATGGGCTTTGGCCGGATAAG
                ********************************* **************************

hab-13c LA1391  ATGGGTATACCCGGCCATTTACAATTCTGCACTTCTGAGAAGTATGAAATTTTTGAACCG
hab-12c LA2863  ATGGGTATACCCGGCCATTTACAATTCTGCACTTCTGAGAAGTATGAAATTTTTGAACCG
hab-13g LA2175  ATGGGTATACCCGGCCATTTACAATTCTGCACTTCTGAGAAGTATGAAATTTTTGAACCG
hab-12g LA2863  ATGGGTATACCCGGCCATTTACAATTCTGCACTTCTGAGAAGTATGAAATTTTTGAACCG
                ************************************************************

hab-13c LA1391  ------------------------------------------------------------
hab-12c LA2863  ------------------------------------------------------------
hab-13g LA2175  GTAAATTACAACGTTATTTAATTTAGCCAATTATCTTAATTTTTGTATTTTTCATTTAAA
hab-12g LA2863  GTAAATTACAACGTTATTTAATTTAGCCAATTATCTTAATTTTTGTATTTTTCATTTAAA

hab-13c LA1391  ---------------------------------------------------------GGT
hab-12c LA2863  ---------------------------------------------------------GGT
hab-13g LA2175  TAGTTTGCATTACTTTTCTGTTAATTTCTAGTAGAAATCATTTACAAATTATAACAGGGT
hab-12g LA2863  TAGTTTGCATTACTTTTCTGTTAATTTCTAGTAGAAATCATTTACAAATTATAACAGGGT
                                                                         ***

hab-13c LA1391  AATGTACTCGATGCTCTGGACCAGCACTGGATTCAGTTGAAGTTTGAACGCGAAGCAGGA
hab-12c LA2863  AATGTACTCGATGCTCTGGACCAGCACTGGATTCAGTTGAAGTTTGAACGCGAAGCAGGA
hab-13g LA2175  AATGTACTCGATGCTCTGGACCAGCACTGGATTCAGTTGAAGTTTGAACGCGAAGCAGGA
hab-12g LA2863  AATGTACTCGATGCTCTGGACCAGCACTGGATTCAGTTGAAGTTTGAACGCGAAGCAGGA
                ************************************************************

hab-13c LA1391  ATCCGTGATCAACCTCTCTGGAGAGATCAATACAAAAAGCATGGAACGTGTTGTTTATCG
hab-12c LA2863  ATCCGTGATCAACCTCTCTGGAGAGATCAATACAAAAAGCATGGAACGTGTTGTTTATCG
hab-13g LA2175  ATCCGTGATCAACCTCTCTGGAGAGATCAATACAAAAAGCATGGAACGTGTTGTTTATCG
hab-12g LA2863  ATCCGTGATCAACCTCTCTGGAGAGATCAATACAAAAAGCATGGAACGTGTTGTTTATCG
```

```
                     ************************************************************

hab-13c LA1391   CGCTACAATCAGTTGCAGTATTTTTTACTAGCCATGCGACTGAAAGAAAAGTTTGATCTT
hab-12c LA2863   CGCTACAATCAGTTGCAGTATTTTTTACTAGCCATGCGACTGAAAGAAAAGTTTGATCTT
hab-13g LA2175   CGCTACAATCAGTTGCAGTATTTTTTACTAGCCATGCGACTGAAAGAAAAGTTTGATCTT
hab-12g LA2863   CGCTACAATCAGTTGCAGTATTTTTTACTAGCCATGCGACTGAAAGAAAAGTTTGATCTT
                 ************************************************************

hab-13c LA1391   TTGACAACTCTCAGAACCCATGGAATTACTCCTGGTACAAAACATACATATAAAAAAATC
hab-12c LA2863   TTGACAACTCTCAGAACCCATGGAATTACTCCTGGTACAAAACATACATATAAAAAAATC
hab-13g LA2175   TTGACAACTCTCAGAACCCATGGAATTACTCCTGGTACAAAACATACATATAAAAAAATC
hab-12g LA2863   TTGACAACTCTCAGAACCCATGGAATTACTCCTGGTACAAAACATACATATAAAAAAATC
                 ************************************************************

hab-13c LA1391   CAAGATGCTATTAAGACAGTCACTCAAGAGGTTCCTGACCTCAAGTGCATTCAATATACT
hab-12c LA2863   CAAGATGCTATTAAGACAGTCACTCAAGAGGTTCCTGACCTCAAGTGCATTCAATATACT
hab-13g LA2175   CAAGATGCTATTAAGACAGTCACTCAAGAGGTTCCTGACCTCAAGTGCATTCAATATACT
hab-12g LA2863   CAAGATGCTATTAAGACAGTCACTCAAGAGGTTCCTGACCTCAAGTGCATTCAATATACT
                 ************************************************************

hab-13c LA1391   AAAGGCGTATTGGAACTATACGAGATAG--------------------------------
hab-12c LA2863   AAAGGCGTATTGGAACTATACGAGATAGGAATATGTTTTACTCCAGAGGCAGATAGTCCA
hab-13g LA2175   AAAGGCGTATTGGAACTATACGAGATAGGAATATGTTTTACTCCAGAGGCAGATAGTCCA
hab-12g LA2863   AAAGGCGTATTGGAACTATACGAGATAGGAATATGTTTTACTCCAGAGGCAGATAGTCCA
                 ****************************

hab-13c LA1391   ------------------------------------------------------------
hab-12c LA2863   TCTCTTTGTCGTCAAAGTAACTCATGCCACCCAACAGAAAACCCGCTGATTTTGTTTCGA
hab-13g LA2175   TCTCTTTGTCGTCAAAGTAACTCATGCCACCCAACAGAAAACCCGCTGATTTTGTTTCGA
hab-12g LA2863   TCTCTTTGTCGTCAAAGTAACTCATGCCACCCAACAGAAAACCCGCTGATTTTGTTTCGA

hab-13c LA1391   ------------------------------------------------------------
hab-12c LA2863   TGATGAATTTCATTGCACATCTTTCCTTTCGGTTTGCTTATGGCTATGTTTAAGAAGATT
hab-13g LA2175   TGATGAATTTCATTGCACATCTTTCCTTTCGGTTTGCTTATGGCT---------------
hab-12g LA2863   TGATGAATTTCATTGCACATCTTT------------------------------------
```

Supplementary Figure 13. Nucleotide sequence alignment of *S. habrochaites* SC-associated *hab-12* S-*RNase* allele with *S. habrochaites* SI-associated *hab-13 S-RNase* allele. *hab-13* sequences from SI individuals in accessions LA2175 (GenBank OK091162) and LA1391 (partial sequence) aligned with *hab-12* allele nucleotide sequences (c= cDNA, g = genomic) in SC accession LA2863 (GenBank OK091161). Potential start codons are highlighted in green, single nucleotide substitution is highlighted in yellow, introns are highlighted in turquoise and stop codons are highlighted in red. Asterisks indicate conservation between all sequences.

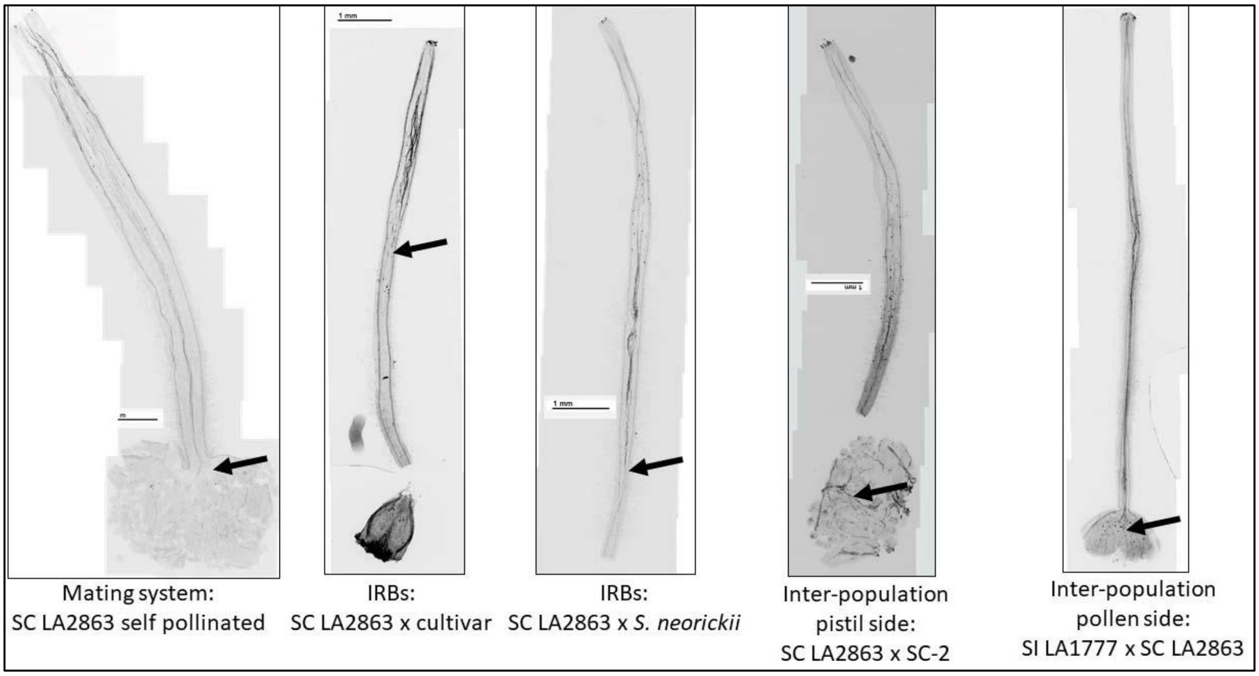

Supplemental Figure 14. Assessing reproductive barriers in the SC-7 group. Mating system, interspecific reproductive barriers (IRBs) and interpopulation barriers were assessed with pollinations as shown with pollen tubes in pistils, stained with Aniline Blue Fluorochrome. Arrows indicate the terminal position of the longest pollen tubes in styles. Signals on each side of the style are vascular bundles.

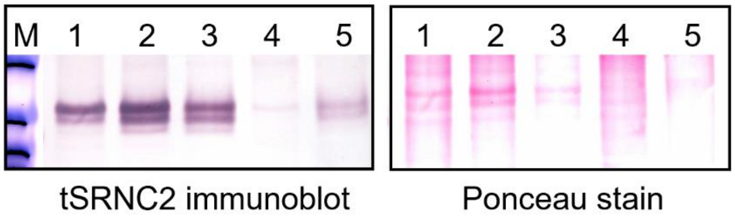

Supplemental Figure 15. S-RNase protein expression in styles of *S. habrochaite*s accessions LA2119 and PI251305. Left side shows immunostaining of stylar extracts using the general S-RNase antibody raised to the conserved C2 region. Right side shows staining of the blot with Ponceau Red as a protein loading control. M = Protein Molecular Weight markers. 1 = LA2119 clone grown in the greenhouse; 2 and 3 = PI25251305 individuals homozygous for the *hab-7* allele, 4 = PI25251305 individual homozygous for the *LhgSRN-1* allele, 5 = PI25251305 individual heterozygous for the *hab-7* and *LhgSRN-1* allele.

References

Broz, A.K., Randle, A.M., Sianta, S.A., Tovar-Méndez, A., Mcclure, B., and Bedinger, P.A. (2017). Mating system transitions in *Solanum habrochaites* impact interactions between populations and species. *New Phytologist* 213**,** 440-454.

Broz, A.K., Simpson-Van Dam, A., Tovar-Méndez, A., Hahn, M.W., Mcclure, B., and Bedinger, P.A. (2021). Spread of self-compatibility constrained by an intrapopulation crossing barrier. *New Phytologist* 231**,** 878-891.

Covey, P.A., Kondo, K., Welch, L., Frank, E., Sianta, S., Kumar, A., Nunez, R., Lopez-Casado, G., Van Der Knaap, E., Rose, J.K., Mcclure, B.A., and Bedinger, P.A. (2010). Multiple features that distinguish unilateral incongruity and self-incompatibility in the tomato clade. *Plant J* 64**,** 367-378.

Landis, J.B., Miller, C.M., Broz, A.K., Bennett, A.A., Carrasquilla-Garcia, N., Cook, D.R., Last, R.L., Bedinger, P.A., and Moghe, G.D. (2021). Migration through a major Andean ecogeographic disruption as a driver of genetic and phenotypic diversity in a wild tomato species. *Molecular Biology and Evolution* 38**,** 3202-3219.
